# Diabetes Risk Perception in Women with a Recent History of Gestational Diabetes Mellitus: A Secondary Analysis from a Belgian Randomized Controlled Trial (MELINDA Study)

**DOI:** 10.3390/jcm14144998

**Published:** 2025-07-15

**Authors:** Yana Vanlaer, Caro Minschart, Ine Snauwaert, Nele Myngheer, Toon Maes, Christophe De Block, Inge Van Pottelbergh, Pascale Abrams, Wouter Vinck, Liesbeth Leuridan, Sabien Driessens, Jaak Billen, Christophe Matthys, Annick Bogaerts, Annouschka Laenen, Chantal Mathieu, Katrien Benhalima

**Affiliations:** 1 Department of Chronic Diseases and Metabolism, Clinical and Experimental Endocrinology, KU Leuven, Herestraat 49, 3000 Leuven, Belgium; yana.vanlaer@kuleuven.be (Y.V.); caro.minschart@hotmail.be (C.M.);; 2 Faculty of Medicine, KU Leuven, Herestraat 49, 3000 Leuven, Belgium; ine.snauwaert@student.kuleuven.be; 3 Department of Endocrinology, General Hospital Groeninge Kortrijk, Campus Kennedylaan 4, 8500 Kortrijk, Belgium; nele.myngheer@azgroeninge.be; 4 Department of Obstetrics & Gynecology, Imelda Hospital, Schoolstraat 55, 2820 Bonheiden, Belgium; toon.maes@imelda.be; 5 Department of Endocrinology-Diabetology-Metabolism, Antwerp University Hospital, Wilrijkstraat 10, 2650 Edegem, Belgium; christophe.deblock@uza.be; 6 Department of Endocrinology, AZORG Aalst, Moorselbaan 164, 9300 Aalst, Belgium; inge.van.pottelbergh@olvz-aalst.be; 7 Department of Endocrinology, ZAS Hospital Sint-Vincentius, Sint-Vincentiusstraat 20, 2018 Antwerpen, Belgium; pascale.abrams@gza.be; 8 Department of Endocrinology, ZAS Hospital Sint-Augustinus, Oosterveldlaan 24, 2610 Wilrijk, Belgium; wouter.vinck@gza.be; 9 Department of Endocrinology, General Hospital Klina, Augustijnslei 100, 2930 Brasschaat, Belgium; liesbeth.leuridan@azturnhout.be (L.L.); sabien.driessens@klina.be (S.D.); 10 Department of Laboratory Medicine, University Hospitals Leuven, Herestraat 49, 3000 Leuven, Belgium; jaak.billen@uzleuven.be; 11 Department of Chronic Diseases and Metabolism, KU Leuven, Herestraat 49, 3000 Leuven, Belgium; christophe.matthys@uzleuven.be; 12 Department of Endocrinology, University Hospitals Leuven, Herestraat 49, 3000 Leuven, Belgium; 13 REALIFE Research Group, Research Unit Woman and Child, Department of Development and Regeneration, KU Leuven, Herestraat 49, 3000 Leuven, Belgium; annick.bogaerts@kuleuven.be; 14 Faculty of Health, University of Plymouth, 3 Portland Mews, Plymouth PL4 8AA, UK; 15 Leuven Biostatistics and Statistical Bioinformatics Centre, KU Leuven, Herestraat 49, 3000 Leuven, Belgium; annouschka.laenen@kuleuven.be

**Keywords:** glucose intolerance, prediabetes, type 2 diabetes mellitus, risk perception, gestational diabetes mellitus

## Abstract

**Background/Objectives:** To evaluate diabetes risk perception in women with prior gestational diabetes mellitus (GDM) and prediabetes in early postpartum. **Methods**: Secondary analysis of a multi-center randomized controlled trial assessing the effectiveness of a mobile-based postpartum lifestyle intervention in women with prediabetes after GDM. Data were collected from the Risk Perception Survey for Developing Diabetes at baseline (6–16 weeks postpartum) and one year post-randomization. Logistic regression was used to analyze the difference between the intervention and control groups on diabetes risk estimation. **Results**: Among 165 women with prediabetes in early postpartum (mean age: 32.1 years, mean BMI: 27.3 kg/m^2^), 58.9% (96) adequately estimated their diabetes risk (moderate or high chance) at baseline. These women smoked less often [2.06% (2) vs. 10.3% (7), *p* = 0.034], reported less anxiety (11.6 ± 3.0 vs. 12.6 ± 3.5, *p* = 0.040), and reported fewer symptoms of depression [30.9% (21) vs. 15.6% (15), *p* = 0.023] compared to women who underestimated their risk. At one year, 58.3% (95) of all women adequately estimated their diabetes risk. In the intervention group, 50.6% (41) adequately estimated their risk at baseline, increasing to 56.8% (46) by the end of the intervention after one year (*p* = 0.638). In the control group, a higher proportion of women adequately estimated their risk at baseline [67.1% (55), (*p* = 0.039)], which decreased to 59.8% (49) at one year (*p* = 0.376), with no significant difference in risk perception between the groups at one year (*p* = 0.638). **Conclusions**: Almost 60% of this high-risk population adequately estimated their diabetes risk, with no significant impact of the lifestyle intervention on risk perception.

## 1. Introduction

The postpartum period following gestational diabetes mellitus (GDM) represents a critical stage in women’s health, where the intersection of metabolic paths and future disease risk unfolds [1,2]. GDM is defined as diabetes diagnosed during pregnancy, provided that overt diabetes was excluded in early pregnancy [3]. GDM is associated with a seven- to tenfold higher risk of developing type 2 diabetes mellitus (T2DM) in the long term [4,5]. Additionally, women with GDM are at greater risk for metabolic syndrome and cardiovascular diseases compared to women without a GDM history [6]. These cardiometabolic conditions, including GDM and T2DM, are increasingly linked to chronic low-grade inflammation—a persistent, mild activation of the immune system. This state is marked by higher levels of inflammatory substances in the blood, such as TNF-α, IL-6, and CRP, which are associated with increased insulin resistance and more β-cell dysfunction, contributing over time to the development of T2DM [7]. During pregnancy, physiological insulin resistance is partially mediated by placental hormones (e.g., human placental lactogen, progesterone, and cortisol). In women with GDM, this insulin resistance is exacerbated by an underlying metabolic vulnerability, including chronic inflammation and genetic predisposition. After delivery, these changes typically resolve, but in women with prediabetes, persistent low-grade inflammation, and incomplete β-cell recovery can drive further dysregulation of glucose regulation, eventually leading to overt T2DM [8].

Most international associations, such as the American College of Obstetricians and Gynecologists (ACOG) and the American Diabetes Association (ADA) recommend screening for T2DM in women through a 75 g oral glucose tolerance test (OGTT) performed between 6 and 16 weeks after delivery [9,10]. Timely screening is essential for early intervention through lifestyle changes or medication [11]. Despite these recommendations, a significant number of women do not undergo T2DM screening during the early postpartum period. This is largely due to insufficient awareness among both women and healthcare providers on their long-term metabolic risk, as well as a lack of adequate long-term follow-up programs [12,13,14,15,16]. This represents a missed opportunity to identify T2DM early and to encourage healthier lifestyles to reduce the risk of its development [14,17].

A potential barrier to postpartum screening and lifestyle changes in women with a GDM history is their low perception of future T2DM risk [18,19]. Studies show that despite awareness of screening recommendations and the risk of progression to T2DM, only 13–16% of women perceive themselves to be at high risk three years post-delivery [20,21].

To reduce the incidence of T2DM among women with a history of GDM, it is crucial to better understand the factors influencing individual risk perception. This is especially important for women with prediabetes in the early postpartum period, representing a particularly a high-risk population with up to a 50% risk of developing T2DM within the next five years [22].

This study aims to assess the diabetes risk perception in women with prediabetes after a recent GDM pregnancy. It is a secondary analysis of the Melinda trial, a large Belgian multi-center randomized controlled trial (RCT) designed to evaluate the effectiveness of a one-year telephone- and mobile-based lifestyle intervention program aimed at promoting healthy living and weight loss in women with prediabetes following GDM [17].

## 2. Materials and Methods

### 2.1. Study Design and Setting

The Melinda trial was a one-year, double-arm, parallel-group, open-label, multi-center RCT to test the efficacy of a telephone- and mobile-based lifestyle intervention program to achieve weight goals in women with prediabetes following a pregnancy with GDM [17].

GDM diagnosis was based on the 2013 World Health Organization (WHO) criteria [23], with treatment following ADA guidelines [10]. If these targets were not met within two weeks of initiating lifestyle measures, insulin was started. Women aged 18 or older, who spoke Dutch, English, or French, and who had prediabetes confirmed by an OGTT between 6 and 16 weeks postpartum, were eligible. Prediabetes was defined as impaired fasting glucose (IFG) [fasting plasma glucose (FPG) 5.6–6.9 mmol/L] and/or impaired glucose intolerance (IGT) (2 h glucose value on the OGTT between 7.8 and 11.0 mmol/L) as recommended by the ADA [10]. Exclusion criteria included overt diabetes mellitus at the postpartum OGTT, the use of glucose-lowering medication other than insulin during pregnancy, and any condition that would hinder participation in a one-year lifestyle program (e.g., serious mental illness, significant language barriers, or plans to relocate) [17].

In short, 240 participants with prediabetes in early postpartum were randomized 1:1, stratified by center and pre-pregnancy BMI, to either the intervention or control group [17]. The intervention arm received a one-year comprehensive lifestyle program, including a face-to-face session, monthly telephone coaching, and the use of the Melinda mobile app to support healthy habits [17]. The face-to-face session focused on education about the long-term health risks associated with GDM and the importance of lifestyle change, and included hands-on training on how to use the app.

The monthly phone calls, conducted by lifestyle coaches trained in motivational interviewing, provided personalized support based on participant progress. The Melinda app included interactive modules on healthy eating and physical activity, consisting of a 12-week dietary coaching program followed by a 12-week physical activity module. In addition, the app provided educational videos and written materials covering topics such as GDM, postpartum health, nutrition, physical activity, breastfeeding, and diabetes risk.

Users could log personal data (e.g., weight, waist circumference, and step counts), set goals, and monitor their progress over time. Physical activity was tracked automatically via integration with a wearable pedometer (Mi Band 2 or 5). The app also included motivational features such as tailored messages, reminders, and progress graphs to enhance user engagement [17].

All participants gave written informed consent in person before randomization. The app did not include a digital informed consent mechanism [17].

Adherence to the app was defined as logging into the app at least once per month during the 12-month intervention period. App usage data were collected automatically and securely [17]. The control group received general guidance on the risks of GDM and recommendations to adopt a healthy lifestyle to prevent type 2 diabetes mellitus (T2DM), with annual primary care follow-up. One year after the 6–16 weeks postpartum OGTT (baseline visit), all participants received another 75 g OGTT with the same examinations as during the baseline visit (one year post-randomization). At baseline and one year post-randomization, all participants underwent a 75 g OGTT. Results from the Melinda trial indicated that while the intervention did not help participants meet weight loss goals, it did reduce the risk of metabolic syndrome and sedentary behavior [16].

The study was registered at ClinicalTrials.gov as NCT03559621, approved by the Medical Ethical Committees of all participating centers (Belgian number: B322201837047), and conducted in accordance with the declaration of Helsinki. Participants provided informed consent before inclusion in the study.

### 2.2. Study Visits and Measurements

Baseline characteristics were collected at the 6–16 week postpartum OGTT visit through clinical exams, questionnaires, blood samples, and medical record review. Both at baseline and one year later, participants underwent a 75 g OGTT. Participants completed self-administered questionnaires at both time points (baseline and one year post-randomization), encompassing a self-designed survey on general habits and socio-economic factors [17], a Food Frequency Questionnaire (FFQ) assessing food and beverage consumption frequency and portion sizes [17], the International Physical Activity Questionnaire (IPAQ) measuring physical activity in different domains, and the Center for Epidemiologic Studies-Depression (CES-D) questionnaire to evaluate symptoms of clinical depression [17]. Additionally, the Spielberger State-Trait Anxiety Inventory (STAI-6) questionnaire was used to measure anxiety levels [17]. At the baseline visit and at the one year post-randomization visit, the Risk Perception Survey for Developing Diabetes (RPS-DD) was completed. The RPS-DD is a validated tool used to assess individuals’ perceived risk of developing T2DM within a specified time frame. This tool has previously also been used in European populations [24]. Therefore, we used the RPS-DD as originally designed, without modification, to ensure its relevance and consistency with the original validation. The RPS-DD questionnaire asks participants to estimate their diabetes risk, providing the following response options: ‘almost no chance’, ‘slight chance’, ‘moderate chance’, and ‘high chance’ [17]. For this secondary analysis, only participants with available data on this questionnaire were included. For this analysis, an adequate estimation of diabetes risk was defined as a self-reported assessment of moderate or high risk, which aligns with established risk classifications for women with a history of GDM [10]. Participants who self-reported their risk as either “moderate” or “high” were therefore considered to have an adequate estimation, while those who reported a “low” risk were considered to have underestimated their actual risk. This threshold was used to determine the alignment between their self-assessed risk and the actual risk based on current clinical guidelines. Women who indicated having ‘almost no chance’, or a ‘slight chance’ to develop T2DM within the next 10 years, were grouped into the group of women who underestimated their future risk of developing T2DM (the underestimator group), while women who indicated to have a ‘moderate chance’, or ‘high chance’ were grouped into the group that adequately estimated their risk to develop T2DM. These categories were made based on the one year post-randomization RPS-DD questionnaire. The RPS-DD questionnaire also included the following questions to assess lifestyle behaviors: “Have you recently made changes in any lifestyle behaviors that you believe will lower your chances of getting diabetes?” and “Are you planning to make changes in any lifestyle behaviors in the near future that you believe will lower your chances of getting diabetes?”. We compared responses to these lifestyle behavior questions across the entire participant cohort and separately for the intervention and control groups, to evaluate the impact of the intervention on participants’ recent and planned lifestyle changes related to diabetes prevention.

### 2.3. Procedures

For the OGTT, women were instructed to fast for at least 10 h, avoid smoking, and refrain from physical activity [17]. At baseline and one year later, glucose and insulin levels were measured fasting and at 30, 60, and 120 min. A fasting lipid profile, including total cholesterol, triglycerides, HDL, and LDL cholesterol, was also assessed, along with HbA1c [17].

Glucose levels were analyzed locally and processed rapidly for diabetes diagnosis, while lipid profile, HbA1c, and insulin analyses were conducted centrally at the Leuven University Hospital for consistency. Plasma glucose was measured using an automated enzymatic method, insulin levels were assessed via immunometric ECLIA (Roche Modular E170), and HbA1c was measured using a Tosoh Glycohemoglobin Analyser (Roche, Basel, Switzerland). Variability in test results was minimal, with 1% for glucose, 6% for insulin, and around 2% for lipids and HbA1c [17].

### 2.4. Statistical Analysis

Descriptive statistics summarized categorical variables as frequencies and percentages, while continuous variables were presented as means with standard deviations (SDs) or medians with interquartile ranges (IQRs), depending on the distribution of the data.

For group comparisons, the Mann–Whitney U test was used for continuous or ordinal variables that were not normally distributed. For categorical variables, either the chi-square test or the Fisher exact test (when expected cell counts were <5) was applied, as appropriate. These choices were made to ensure accurate testing based on the nature and distribution of each variable.

Paired binary data were analyzed using the McNemar test. To assess the effect of the intervention on diabetes risk perception, logistic regression was used, adjusting for baseline values (adequate risk estimation at baseline). Results are presented as adjusted odds ratios (ORs) with 95% confidence intervals (CIs).

A two-sided significance level of 0.05 was applied to all statistical tests. Given the exploratory nature of this secondary analysis, no adjustments for multiple comparisons were performed. All statistical analyses were conducted by statistician A. Laenen using SAS software (version 9.4 for Windows, 2023).

## 3. Results

**Figure 1:**
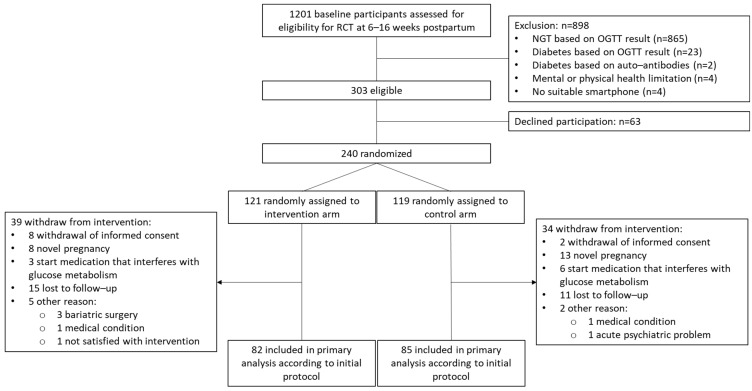
Flowchart of participants included in the Melinda study. RCT, randomized controlled trial; NGT, normal glucose tolerance; OGTT, oral glucose tolerance test.

**Figure 2:**
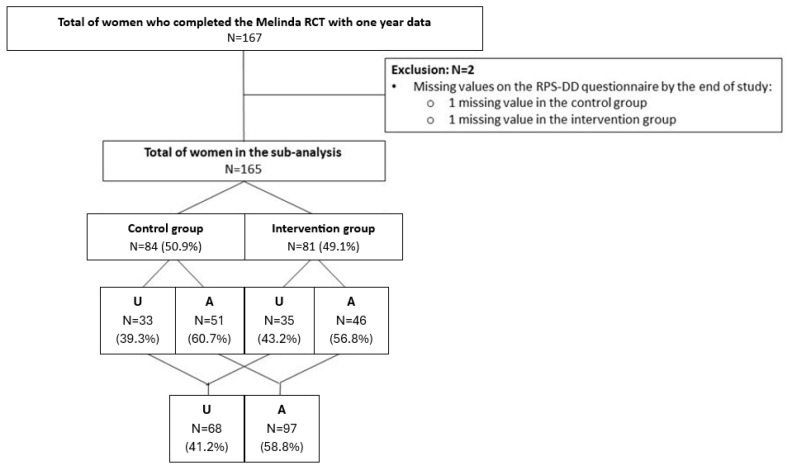
Flowchart of participants included in the secondary analysis. RCT, randomized controlled trial; RPS-DD questionnaire, risk perception survey for developing diabetes questionnaire; U, underestimated the risk to develop T2DM (underestimator group: almost no chance and slight chance); A, adequately estimated the risk to develop T2DM (moderate chance and high chance).

Of the 240 randomized participants in the Melinda RCT, 167 completed the study and were included in the final analysis of the Melinda trial (82 in the intervention group and 85 in the control group). A total of 73 participants (39 in the intervention group and 34 in the control group) withdrew from the study, resulting in a dropout rate of 30.4%. The reasons for dropout have been previously described (11) (Figure 1). Participants who stopped early were less likely to underestimate their diabetes risk at baseline compared to those who completed the 1-year follow-up [26.0% (19) vs. 40.6% (67), *p* = 0.040] (Supplementary Table S1). Women without data on the RPS-DD questionnaire were excluded (a total of two women), leaving 165 women in this secondary analysis (Figure 2). Due to two missing values at baseline on the RPS-DD questionnaire, pairwise comparisons were performed solely on patients with complete data at both baseline and one year postpartum.

**Figure 3:**
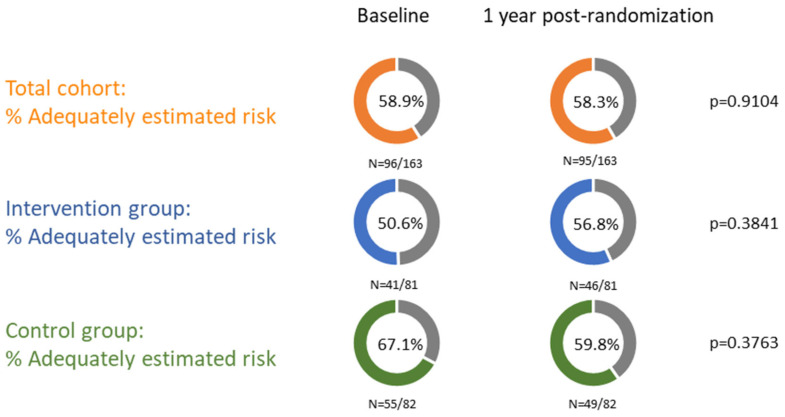
Evaluation of diabetes risk perception from baseline (6–16 weeks postpartum) to one year after randomization. The McNemar test was used for the analysis of paired binary data. Data were paired for a total of 163 women. This is a summary of the percentage of women who adequately estimated their diabetes risk at baseline and at one year after randomization in the whole cohort, intervention group and control group.

Among 165 women with prediabetes in early postpartum, the mean age was 32.0 ± 4.1 years and the mean BMI was 27.3 ± 5.5 kg/m^2^. Overall, 58.9% (96) adequately estimated their diabetes risk at baseline (Figure 3). These women smoked less often [2.06% (2) vs. 10.3% (7), *p* = 0.034], reported less anxiety (11.6 ± 3.0 vs. 12.6 ± 3.5, *p* = 0.040), and had symptoms of depression less often [30.9% (21) vs. 15.6% (15), *p* = 0.023] at baseline compared to women who underestimated their diabetes risk. The education level, history of GDM, pre-pregnancy BMI, and family history of T2DM showed no significant impact on the diabetes risk estimation (Table 1). At one year, 58.3% (95) of the whole cohort adequately estimated their risk. There were no significant differences in characteristics at one year between women who underestimated their risk and women who adequately estimated their diabetes risk, except that the group who adequately estimated their risk lost less weight at one year postpartum (−2.3 ± 6.3 vs. 0.1 ± 4.9, *p* = 0.025) (Table 1, Figure 3).

Women in the control group were older (32.8 ± 4.09 vs. 31.4 ± 4.15, *p* = 0.018) compared to the intervention group (Supplementary Table S2). In the control group, women who underestimated their risk reported higher anxiety levels compared to women who adequately estimated their risk (STAI-6 scores: 12.9 ± 3.4 vs. 11.2 ± 2.9, *p* = 0.014) at baseline. In the intervention group, women who underestimated their risk were less often highly educated [60% (21) vs. 83% (38), *p* = 0.004], more often single [29% (10) vs. 9% (4), *p* = 0.035], and had a higher pre-pregnancy BMI (27.7 ± 5.0 vs. 25.3 ± 5.2 kg/m^2^, *p* = 0.022) compared to women who adequately estimated their risk (Table 2, Table 3 and Table 4).

At baseline, more women in the control group adequately estimated their risk compared to the intervention group [67.1% (55) vs. 50.6% (41), *p* = 0.039]. However, at one year, the number of women who adequately estimated their diabetes risk decreased in the control group to 59.8% (49) (*p* = 0.376) and increased in the intervention group to 56.8% (46) (*p* = 0.384), with no significant difference between the control and intervention group in the number of women who adequately estimated their diabetes risk at one year, as assessed using logistic regression (adjusted odds ratio 0.88, 95% CI 0.47–1.66; *p* = 0.699) (Table 4, Figure 3, Supplementary Tables S2–S4). Exploratory analyses suggest that improvements in diabetes risk perception may be more pronounced in groups with higher education and middle income (Supplementary Tables S5 and S6).

Participants were asked about recent and planned changes in lifestyle behaviors to reduce their diabetes risk. In the whole cohort at one year, 51.5% (85) indicated that they made recent changes, and 77.6% (128) planned future changes in lifestyle. Among women who underestimated their diabetes risk, 54.4% (37) reported that they made recent changes, and 67.7% (46) planned future changes. Of those who adequately estimated their risk, 49.5% (48) reported making recent changes and 84.5% (82) planned future changes (*p* = 0.014). In the intervention group, the underestimator group more often reported making changes in lifestyle compared to women who adequately estimated their risk for making recent changes [62.9% (22) vs. 39.1% (18), *p* = 0.045], while in the control group, the underestimator group less often reported planning changes in lifestyle in the future (57.6% (19) in the underestimator group vs. 84.3% (43) who adequately estimated their risk, *p* = 0.010). There were no significant differences between the intervention and control group regarding their recent changes (*p* = 0.642) and planned changes (*p* = 0.266) in lifestyle behaviors to reduce their diabetes risk (Table 1, Table 2 and Table 3).

## 4. Discussion

Our findings indicate that about 40% of this high-risk population underestimates their risk of developing diabetes in the future. Although the number of women who adequately estimated their diabetes risk increased after one year in the group who received the blended mobile-based lifestyle program and decreased in the usual care group, the lifestyle program did not significantly improve the diabetes risk estimation. Therefore, this study highlights the importance of assessing and addressing diabetes risk perception, particularly in the postpartum period, as an appropriate diabetes risk perception might help to improve adherence to lifestyle interventions and attendance to diabetes screening programs. Women who adequately estimated their diabetes risk were more likely to make and plan lifestyle changes, while those who underestimated their risk were less likely to plan these changes. Despite the intervention, which aimed to improve risk perception through mobile-based lifestyle support, the results suggest that factors such as education level, socioeconomic status, and psychological factors may play an important role in shaping diabetes risk perception.

In this study, an adequate estimation of diabetes risk was defined as a self-reported assessment of moderate or high risk, which aligns with established risk classifications for women with a history of GDM [25,26]. Participants who self-reported their risk as either “moderate” or “high” were considered to have an adequate estimation, while those who reported a “low” risk were considered to have underestimated their actual risk. This threshold was used to determine the alignment between their self-assessed risk and the actual risk based on current clinical guidelines. There are limited data on how women with prediabetes in the early postpartum period perceive their diabetes risk after experiencing GDM. This population faces a significant risk, with up to 50% developing T2DM within five years [22]. This study provides novel insights into the diabetes risk perception of this group and examines whether a mobile-based lifestyle intervention can improve their understanding of this risk.

Our findings reveal that approximately 40% of women underestimated their risk of developing T2DM within 10 years. Factors such as educational level, GDM history, pre-pregnancy BMI, and family history of T2DM did not significantly influence their risk perception. While more women in the intervention group estimated their diabetes risk more adequately after one year, the difference compared to the control group was not statistically significant. This suggests that the mobile-based lifestyle intervention did not meaningfully enhance awareness of their high T2DM risk. We also examined whether women from lower socioeconomic backgrounds benefited from the intervention. Our analysis suggests that women with higher education and middle incomes showed greater improvements in risk perception, indicating that alternative approaches may be required to better engage women of lower socioeconomic status. Similar studies with lower-risk GDM populations found lifestyle interventions had little impact on risk perception, possibly because these programs focus more on behavior change than on addressing cognitive or emotional factors [21,26]. Another potential explanation why women with a history of GDM underestimate their long-term risk for T2DM, might be the fact that they do not feel ill as early signs of metabolic dysfunction do not give any symptoms [3].

Several factors may explain the lack of significant impact on risk perception. Women from lower socioeconomic backgrounds and with lower education levels were less likely to benefit from the intervention, indicating that more targeted approaches are needed for these groups [15,21]. Additionally, the absence of symptoms in women with a history of GDM could lead them to underestimate their risk, as early signs of metabolic dysfunction are often asymptomatic [3]. Psychosocial factors such as anxiety, stress, and the sense of control over health may also play a role in shaping risk perception. Interventions that address both physical health and mental well-being, including anxiety management, might be more effective in improving risk perception [25,27].

Although our study did not directly evaluate whether improving risk perception prevents T2DM, existing research indicates that increased awareness can drive behavior changes, such as adopting healthier diets and increasing physical activity. Future studies should explore whether improved risk perception can directly prevent T2DM development [28].

The postpartum period presents particular challenges for women trying to achieve lasting behavior changes due to time constraints, stress, and fatigue [29,30]. Our findings highlight the difficulty of altering risk perception in postpartum women using lifestyle interventions. Longer-term support may be necessary, as sustained behavior changes often require extended reinforcement [25].

Future interventions are needed to adopt a more comprehensive and personalized approach to effectively alter the diabetes risk perception [21,26]. Our results suggest that sociodemographic factors significantly influence diabetes risk perception. Women who adequately estimated their risk were less likely to smoke, had fewer symptoms of anxiety and depression, and tended to have higher education levels, be in a relationship, and have a lower pre-pregnancy BMI compared to those who underestimated their risk. These findings are consistent with previous studies showing that factors like education, income, ethnicity, and healthcare access affect individuals’ health risk perception. These findings underscore the need for targeted and culturally sensitive interventions to improve diabetes risk perception and prevention efforts [15]. In the intervention group, women from lower socioeconomic backgrounds were more likely to underestimate their diabetes risk, a pattern not observed in the control group. This may have occurred by chance, as randomization did not account for risk perception. Social support emerged as a key factor in the success of lifestyle interventions. Women with lower education levels or single status may have had less access to social support, making it more difficult to sustain healthy behaviors. Tailored interventions that enhance social support for these groups could improve health outcomes [27]. Diabetes risk perception involves both physical and psychological aspects. Anxiety, stress, and a sense of control can influence how women perceive their risk [31,32]. Interventions incorporating mental health support, such as anxiety management, may prove more effective in improving risk perception [15,32]. Although individual therapy may slightly outperform group therapy in treating depression, group therapy remains a valuable and cost-effective option, particularly in settings where social support is important [33].

Women who underestimated their risk were less likely to plan lifestyle changes compared to those who adequately assessed their diabetes risk. Interestingly, in the intervention group, those who underestimated their risk reported making more lifestyle changes than those who adequately estimated their risk. In contrast, the proportion of women with adequate risk perception declined in the control group over the course of one year. A possible explanation for this decrease is the lack of structured follow-up or reinforcement of health messages after the early postpartum period in the control group. As time progresses, women’s focus may shift toward family and work responsibilities, reducing the awareness on the long-term health risks associated with GDM. Without continued engagement from healthcare providers, risk awareness may fade, leading to an underestimation of diabetes risk over time [19,34]. However, no significant differences were found between the two groups in dietary intake or physical activity, suggesting a potential bias in self-reporting. It is also possible that women who made lifestyle changes believed they had reduced their diabetes risk, and were therefore more likely to underestimate that risk.

This study has several strengths. It provides longitudinal data on diabetes risk perception among women with prediabetes in the early postpartum period and evaluates the impact of a mobile-based lifestyle intervention on this perception. This study has also several important limitations. First, the final sample size was relatively modest, which may have reduced the statistical power to detect differences, particularly in subgroup analyses. As this was a secondary and exploratory analysis, the study was not specifically powered to detect changes in risk perception. Second, challenges in follow-up, including participant drop-out and varying degrees of engagement with the intervention, may have introduced bias. Although adherence to the Melinda app was defined as logging in at least once per month, actual engagement levels may have varied widely across participants, and not all users may have interacted with all app features equally. Third, the intervention relied primarily on digital tools and remote coaching. The limited involvement of in-person contact or individualized counseling by certified healthcare professionals—such as dietitians, psychologists, or endocrinologists—may have reduced the depth and personalization of behavioral support. This could have influenced both the perception of diabetes risk and the overall effectiveness of the intervention. Finally, the predominantly Caucasian study population limits the generalizability of our findings to more ethnically diverse groups and healthcare settings.

## 5. Conclusions

In conclusion, nearly 60% of all participants adequately estimated their risk to develop diabetes, with no significant difference between those who received a one-year blended-care lifestyle intervention (intervention group) and those who received usual care (control group). This underscores the importance of addressing diabetes risk perception in postpartum women with a history of GDM. Tailored interventions are needed to target individuals who underestimate their risk and to address psychological and social factors. Future research should focus on developing comprehensive interventions that incorporate psychological support, enhance social support, and provide long-term reinforcement to sustain behavior change.

## Figures and Tables

**Table 1:** Comparison of the underestimator group with the group who adequately estimated their risk for diabetes from the whole cohort.

	Underestimator Group (N = 68, 41.2%) ^d,e^	Group That Adequately Estimated the Risk (N = 97, 58.8%) ^d,e^	*p*-Value ^f^
**General characteristics ^a^**			
Age (years) baseline	32.2 ± 4.69	32.0 ± 3.79	0.775
% Non-Caucasian	19.1 (13)	15.5 (15)	0.536
Highest education			0.158
%None/Primary school	0.00 (0)	1.03 (1)	
%Until age of 15 years	11.76 (8)	4.12 (4)	
%High school	19.12 (13)	15. 46 (15)	
%Higher education (bachelor/master)	69.12 (47)	79. 38 (77)	
% Paid professional activity	80.88 (55)	89.69 (87)	0.117
Monthly net income family			0.197
%Low income EUR < 1500	4.41 (3)	3.13 (3)	
% EUR 1500–5000	91. 18 (62)	84. 38 (81)	
% > EUR 5000	4.41 (3)	12. 50 (12)	
% Living without partner	23.53 (16)	13.40 (13)	0.101
% Currently smoking	10.29 (7)	2.06 (2)	**0.034**
% Multiparity	50.00 (34)	54.64 (53)	0.635
% History of GDM in previous pregnancy	24.39 (10)	20.00 (13)	0.634
% History of PCOS	6.25 (4)	4.30 (4)	0.716
% History of miscarriage	33.82 (23)	35.05 (34)	1.000
Pre-pregnancy BMI (kg/m^2^)	27.2 ± 5.35	26.8 ± 5.53	0.556
% First degree family history of T2DM	30.77 (20)	28.26 (26)	0.859
% Second degree family history of T2DM	56.14 (32)	65.48 (55)	0.293
% Insulin use in pregnancy	32.35 (22)	32.99 (32)	1.000
Insulin injection per day in pregnancy			0.643
% 1×/day	13.64 (2)	6.25 (2)	
% 3×/day	9.09 (2)	18.75 (6)	
% Long-acting insulin	27.27 (6)	31.25 (10)	
%Combination meal insulin + long-acting insulin	50.00 (11)	43.75 (14)	
**Baseline (6–16 weeks postpartum) ^b^**			
FPG (mmol/L)	5.21 ± 0.58	5.27 ± 0.59	0.430
Glycemia 60 min (mmol/L)	9.83 ± 1.93	9.24 ± 1.99	**0.018**
Glycemia 120 min (mmol/L)	8.42 ± 1.46	7.98 ± 1.36	**0.018**
Timing OGTT (weeks)	12.1 ± 2.91	12.7 ± 2.71	0.078
BMI (kg/m^2^)	27.4 ± 5.49	27.2 ± 5.60	0.650
Mean systolic blood pressure (mmHg)	118.0 ± 12.59	118.3 ± 11.64	0.948
Mean diastolic blood pressure (mmHg)	74.4 ± 9.68	75.7 ± 8.81	0.430
% Hypertension	10.29 (7)	7.22 (7)	0.574
Waist circumference (cm)	90.9 ± 12.58	91.5 ± 13.64	0.604
PPWR (kg)	0.6 ± 5.23	1.0 ± 4.47	0.378
PPWR > 0 kg	51.47 (35)	56.70 (55)	0.529
PPWR > 5 kg	16.18 (11)	12.37 (12)	0.502
HbA1c (%)	5.4 ± 0.35	5.4 ± 0.31	0.802
HbA1c (mmol/mol)	35.5 ± 3.83	35.5 ± 3.41	0.802
Fasting LDL-cholesterol (mmol/L)	2.96 ± 0.83	2.91 ± 0.77	0.847
FFQ:			
Total water (g)	2146.6 (1789.2–2489.3)	2154.4 (1670.4–2462.8)	0.735
Total fruit (g)	108.3 (51.5–225.0)	96.5 (51.5–225.0)	0.685
Total vegetables (g)	240.0 (120.0–240.0)	240.0 (120.0–240.0)	0.414
Total meat (g)	108.6 (68.5–140.5)	114.3 (76.2–149.9)	0.742
Total fish (g)	15.8 (8.6–28.6)	16.4 (8.9–27.5)	0.633
Total rest group (g)	211.7 (89.9–483.5)	254.9 (86.4–476.0)	0.725
Daily protein intake (g)	62.8 (52.2–78.0)	62.2 (49.9–74.5)	0.481
Daily fat intake (g)	50.5 (40.8–69.6)	52.8 (43.0–64.3)	0.951
Daily carbohydrate intake (g)	189.1 (146.7–217.3)	173.7 (146.8–220.9)	0.538
Daily fiber intake (g)	18.0 (14.6–21.4)	18.1 (13.0–21.8)	0.486
IPAQ/METs category at time of OGTT			0.423
% Low	9.38 (6)	16.13 (15)	
% Moderate	46.88 (30)	39.78 (37)	
% High	43.75 (28)	44.09 (41)	
STAI-6	12.6± 3.51	11.6 ± 2.95	**0.040**
% Clinical depression (≥16 on CES-D questionnaire)	30.88 (21)	15.63 (15)	**0.023**
**One year after randomization ^c^**			
% Breastfeeding	73.53 (50)	80.21 (77)	0.346
FPG (mmol/L)	5.27 ± 0.75	5.35 ± 0.59	0.174
Glycemia 60 min (mmol/L)	8.91 ± 2.51	8.86 ± 2.23	0.988
Glycemia 120 min (mmol/L)	7.38 ± 2.31	7.25 ± 1.86	0.831
Timing OGTT (weeks)	65.0 ± 3.88	64.9 ±3.39	0.880
% T2DM	5.88 (4)	4.12 (4)	0.723
% IFG	21.21 (7)	29.31 (17)	
% IGT	48.48 (16)	44.83 (26)	
% IFG + IGT	30.30 (10)	25.86 (15)	
BMI (kg/m^2^)	26.3 ± 5.63	26.8 ± 6.00	0.678
Mean systolic blood pressure (mmHg)	119.0 ± 14.74	118.7 ± 11.20	0.813
Mean diastolic blood pressure (mmHg)	76.1 ± 11.27	77.4 ± 9.60	0.580
% Hypertension	16.18 (11)	13.40 (13)	0.658
Waist circumference (cm)	86.9 ± 15.23	88.17 ± 14.18	0.399
PPWR (kg)	−2.31 ± 6.291	0.13 ± 4.847	**0.025**
PPWR > 0 kg	38.24 (26)	48.45 (47)	0.207
PPWR > 5 kg	7.35 (5)	13.40 (13)	0.311
HbA1c (%)	5.3 ± 0.42	5.3 ± 0.31	0.662
HbA1c (mmol/mol)	35.0 ± 4.58	34.7 ± 3.33	0.662
Fasting LDL-cholesterol (mmol/L)	2.81 ± 0.84	2.91 ± 0.85	0.290
FFQ:			
Total water (g)	1884.0 (1512.1–2238.4)	2125.1 (1617.9–2406.2)	0.193
Total fruit (g)	120.0 (51.5–225.0)	96.5 (51.5–225.0)	0.584
Total vegetables (g)	240.0 (120.0–240.0)	240.0 (120.0–240.0)	0.848
Total meat (g)	89.8 (63.9–137.6)	91.1 (67.0–135.3)	0.857
Total fish (g)	15.4 (8.2–23.8)	15.0 (9.6–22.5)	0.734
Total rest group (g)	161.2 (54.9–461.3)	209.5 (75.9–430.5)	0.548
Daily protein intake (g)	56.5 (42.4–69.1)	55.0 (45.6–66.4)	0.963
Daily fat intake (g)	46.8 (33.5–62.3)	45.6 (37.5–56.0)	0.883
Daily carbohydrate intake (g)	148.8 (124.5–189.2)	154.5 (129.2–185.2)	0.791
Daily fiber intake (g)	15.8 (12.4–19.8)	16.0 (12.4–19.6)	0.846
IPAQ/METs category at time of OGTT			0.275
% Low	5.26 (3)	9.09 (8)	
% Moderate	33.33 (19)	43.18 (38)	
% High	61.40 (35)	47.73 (42)	
STAI-6	12.8 ± 3.78	12.4 ± 3.56	0.445
% Clinical depression (≥16 on CES-D questionnaire)	30.88 (21)	23.71 (23)	0.372
% Recent changes to reduce risk	54.41 (37)	49.48 (48)	0.635
% Planning changes to reduce risk	67.65 (46)	84.54 (82)	**0.014**

^a^ GDM, gestational diabetes mellitus; PCOS, polycystic ovary syndrome; BMI, body mass index; and T2DM, type 2 diabetes mellitus. ^b^ FPG, fasting plasma glucose; OGTT, oral glucose tolerance test; PPWR, postpartum weight retention; LDL-cholesterol, low-density lipoprotein cholesterol; FFQ, Food Frequency Questionnaire; IPAQ, International Physical Activity Questionnaire; METs, metabolic equivalent of task [MET] minutes/week; CES-D, Center for Epidemiologic Studies–Depression; and STAI-6, Spielberger State-Trait Anxiety Inventory. ^c^ IFG, impaired fasting glucose; IGT, impaired glucose tolerance. ^d,e^ Categorical variables were analyzed using Fisher’s exact test and are presented as frequencies % (n); continuous variables were analyzed using the Mann–Whitney U test and are presented as mean ± SD if normally distributed and as median ± IQR if not normally distributed. ^f^ Differences are considered significant at *p*-value < 0.05. Bold means a statistically significant value of *p* < 0.05.

**Table 2:** Comparison of the underestimator group with the group who adequately estimated their risk for diabetes from the intervention group.

	Underestimator Group (N = 35, 43.21%) ^d,e^	Group That Adequately Estimated the Risk (N = 46, 56.79%) ^d,e^	*p*-Value ^f^
**General characteristics ^a^**			
Age (years)	30.7 ± 4.57	31.8 ± 3.79	0.284
% Non-Caucasian	17.14 (6)	10.87 (5)	0.518
Highest education			**0.004**
%Until age of 15 years	20.00(7)	0.00 (0)	
%High school	20.00(7)	17.39 (8)	
%Higher education (bachelor/master)	60.00 (21)	82.61 (38)	
% Paid professional activity	85.71 (30)	91.30 (42)	0.490
Monthly net income family			0.066
%Low income EUR <1500	2.86 (1)	0.0(0)	
% EUR 1500–5000	94.29 (33)	84.78 (39)	
% > EUR 5000	2.86 (1)	15.22 (7)	
% Living without partner	28.57 (10)	8.70 (4)	**0.035**
% Currently smoking	8.57 (3)	0.00 (0)	0.077
% Multiparity	51.43 (18)	47.83 (22)	0.824
% History of GDM in previous pregnancy	25.00 (5)	18.52 (5)	0.723
% History of PCOS	9.09 (3)	4.44 (2)	0.645
% History of miscarriage	25.71 (9)	26.09 (12)	1.000
Pre-pregnancy BMI (kg/m^2^)	27.7 ± 4.95	25.3 ± 5.19	**0.022**
% First degree family history of T2DM	24.24 (8)	25.00 (11)	1.000
% Second degree family history of T2DM	53.57 (15)	62.79 (27)	0.469
**Baseline (6–16 weeks postpartum) ^b^**			
FPG (mmol/L)	5.21 ± 0.58	5.16 ± 0.58	0.764
Glycemia 60 min (mmol/L)	9.87 ± 1.94	9.12 ± 2.17	**0.033**
Glycemia 120 min (mmol/L)	8.33 ± 1.79	8.16 ± 126	0.249
Timing OGTT (weeks)	12.2 ± 3.32	12.4 ± 2.89	0.410
BMI (kg/m^2^)	27.7 ± 538	25.76 ± 5.24	0.089
Mean systolic blood pressure (mmHg)	116.7 ± 13.21	117.5 ± 10.53	0.786
Mean diastolic blood pressure (mmHg)	75.3 ± 8.62	74.9 ± 7.21	0.834
% Hypertension	8.57 (3)	4.35 (2)	0.647
Waist circumference (cm)	90.8 ± 13.24	87.6 ± 12.04	0.360
PPWR (kg)	−0.1 ± 5.41	1.26 ± 3.88	0.226
PPWR > 0 kg	45.71 (16)	60.87 (28)	0.187
PPWR > 5 kg	17.14 (6)	10.87 (5)	0.518
HbA1c (%)	5.4 ± 030	5.4 ± 0.22	0.826
HbA1c (mmol/mol)	35.3 ± 3.26	35.6 ± 2.38	0.826
Fasting LDL-cholesterol (mmol/L)	2.81 ± 0.77	2.89 ± 0.71	0.551
FFQ:			
Total water (g)	2149.4 (1789.2–2507.3)	2042.0 (1653.8–2498.7)	0.478
Total fruit (g)	96.5 (51.5–225.0)	96.5 (51.5–225.0)	0.749
Total vegetables (g)	240.0 (120.0–360.0)	240.0 (120.0–240.0)	0.129
Total meat (g)	122.1 (72.5–173.6)	115.2 (82.2–150.8)	0.808
Total fish (g)	11.8 (7.0–27.5)	18.5 (8.6–29.7)	0.256
Total rest group (g)	230.1 (94.3–496.2)	262.8 (92.8–493.0)	0.966
Daily protein intake (g)	67.2 (51.7–77.1)	60.5 (49.6–77.8)	0.267
Daily fat intake (g)	50.1 (40.3–70.5)	48.7 (42.3–57.9)	0.514
Daily carbohydrate intake (g)	194.0 (141.0–220.6)	171.6 (140.0–216.7)	0.288
Daily fiber intake (g)	17.9 (15.5–23.5)	18.3 (11.4–21.5)	0.243
IPAQ/METs category at time of OGTT			0.325
% Low	5.88(2)	17.39 (8)	
% Moderate	47.06 (16)	41.30 (19)	
% High	47.06 (16)	41.30 (19)	
STAI-6	12.3 ± 3.59	11.9 ± 3.01	0.611
**One year after randomization ^c^**			
% Breastfeeding	65.71 (23)	76.09 (35)	0.330
FPG (mmol/L)	5.18 ± 0.53	5.22 ± 0.50	0.569
Glycemia 60 min (mmol/L)	8.93 ± 2.46	9.06 ± 2.29	0.797
Glycemia 120 min (mmol/L)	7.30 ± 1.83	7.14 ± 2.17	0.703
Timing OGTT (weeks)	64.1 ± 3.70	64.4 ± 3.330	0.357
% T2DM	2.86 (1)	4.35 (2)	0.844
% IFG	17.65 (3)	26.92 (7)	
% IGT	52.94 (9)	50.00 (13)	
% IFG + IGT	29.41 (5)	23.08 (6)	
BMI (kg/m^2^)	26.5 ± 5.30	25.1 ± 5.58	0.182
Mean systolic blood pressure (mmHg)	119.8 ± 14.42	117.6 ± 11.30	0.590
Mean diastolic blood pressure (mmHg)	76.1 ± 12.92	78.4 ± 9.89	0.567
% Hypertension	17.14 (6)	15.22 (7)	1.000
Waist circumference (cm)	87.1 ± 13.00	84.1 ± 13.54	0.303
PPWR (kg)	−3.2 ± 5.97	−0.4 ± 4.43	0.088
PPWR > 0 kg	34.29 (12)	43.48 (20)	0.493
PPWR > 5 kg	5.71 (2)	8.70 (4)	0.694
HbA1c (%)	5.3 ± 0.27	5.3 ± 0.25	0.876
HbA1c (mmol/mol)	34.6 ± 2.93	34.6 ± 2.76	0.876
Fasting LDL-cholesterol (mmol/L)	2.69 ± 0.80	2.88 ± 0.80	0.172
FFQ:			
Total water (g)	1989.9 (1570.7–2254.9)	1828.5 (1568.8–2318.6)	0.757
Total fruit (g)	120.0 (51.5–225.0)	95.4 (51.5–225.0)	0.255
Total vegetables (g)	240.0 (120.0–240.0)	240.0 (120.0–240.0)	0.704
Total meat (g)	92.3 (63.9–141.1)	88.8 (69.7–127.7)	0.797
Total fish (g)	14.5 (7.0–23.8)	16.6 (10.8–27.5)	0.254
Total rest group (g)	171.2 (60.4–346.4)	198.0 (56.9–304.4)	0.996
Daily protein intake (g)	57.6 (42.3–69.9)	54.5 (44.1–64.5)	0.678
Daily fat intake (g)	41.9 (32.0–62.8)	43.7 (36.8–55.1)	0.966
Daily carbohydrate intake (g)	146.2 (124.4–179.2)	144.6 (120.5–178.2)	0.886
Daily fiber intake (g)	15.7 (11.4–20.6)	16.0 (13.5–18.6)	0.901
IPAQ/METs category at time of OGTT			0.778
% Low	0.00(0)	2.33 (1)	
% Moderate	37.93 (11)	44.19 (19)	
% High	62.07 (18)	53.49 (23)	
% Clinical depression (≥16 on CES-D questionnaire)	31.43 (11)	30.43 (14)	1.000
STAI-6	12.6 ± 3.85	12.3 ± 3.63	0.571
% Recent changes to reduce risk	62.86 (22)	39.13 (18)	**0.045**
% Planning changes to reduce risk	77.14 (27)	84.78 (39)	0.402

^a^ GDM, gestational diabetes mellitus; PCOS, polycystic ovary syndrome; BMI, body mass index; and T2DM, type 2 diabetes mellitus. ^b^ FPG, fasting plasma glucose; OGTT, oral glucose tolerance test; PPWR, postpartum weight retention; LDL-cholesterol, low-density lipoprotein cholesterol; FFQ, Food Frequency Questionnaire; IPAQ, International Physical Activity Questionnaire; METs, metabolic equivalent of task [MET] minutes/week; CES-D, Center for Epidemiologic Studies–Depression; and STAI-6, Spielberger State-Trait Anxiety Inventory. ^c^ IFG, impaired fasting glucose; IGT, impaired glucose tolerance. ^d,e^ Categorical variables were analyzed using Fisher’s exact test and are presented as frequencies % (n); continuous variables were analyzed using the Mann–Whitney U test and are presented as mean ± SD if normally distributed and as median ± IQR if not normally distributed. ^f^ Differences are considered significant at *p*-value < 0.05. Bold means a statistically significant value of *p* < 0.05.

**Table 3:** Comparison of the underestimator group with the group who adequately estimated their risk for diabetes from the control group.

	Underestimator Group (N = 33, 39.29%) ^d,e^	Group That Adequately Estimated the Risk (N = 51, 60.71%) ^d,e^	*p*-Value ^f^
**General characteristics ^a^**			
Age (years)	33.7 ± 4.39	32.2 ± 3.83	0.119
% Non-Caucasian	21.21 (7)	19.61 (10)	1.000
Highest education			0.822
%None/primary school	0.00 (0)	1.96 (1)	
%Until age of 15 years	3.03 (1)	7.84 (4)	
%High school	18.18 (6)	13.73 (7)	
%Higher education (bachelor/master)	78.79 (26)	76.47 (39)	
% Paid professional activity	75.76 (25)	88.24 (45)	0.148
Monthly net income family			0.892
%Low income EUR <1500	6.06 (2)	6.00 (3)	
% EUR 1500–5000	87.88 (29)	84.00 (42)	
% > EUR 5000	6.06 (2)	10.00 (5)	
% Living without partner	18.18 (6)	17.65 (9)	1.000
% Currently smoking	12.12 (4)	3.92 (2)	0.205
% Multiparity	48.48 (16)	60.78 (31)	0.368
% History of GDM in previous pregnancy	23.81 (5)	21.05 (8)	1.000
% History of PCOS	3.23 (1)	4.17 (2)	1.000
% History of miscarriage	42.42 (14)	43.14 (22)	1.000
Pre-pregnancy BMI (kg/m^2^)	26.6 ± 5.77	28.1 ± 5.53	0.192
% First degree family history of T2DM	37.50 (12)	31.25 (15)	0.633
% Second degree family history of T2DM	58.62 (17)	68.29 (28)	0.454
**Baseline (6–16 weeks postpartum) ^b^**			
FPG (mmol/L)	5.19 ± 0.58	5.37 ± 0.58	0.191
Glycemia 60 min (mmol/L)	9.75 ± 1.95	9.32 ± 1.82	0.282
Glycemia 120 min (mmol/L)	8.50 ± 1.00	7.83 ± 1.44	**0.029**
Timing OGTT (weeks)	12.0 ± 2.44	12.9 ± 2.53	0.114
BMI (kg/m^2^)	27.1 ± 5.67	28.4 ± 5.66	0.329
Mean systolic blood pressure (mmHg)	119.3 ± 11.95	119.1 ± 12.61	0.869
Mean diastolic blood pressure (mmHg)	73.4 ± 10.74	76.3 ± 10.07	0.224
% Hypertension	12.12 (4)	9.80 (5)	0.733
Waist circumference (cm)	90.9 ± 12.03	95.2 ± 14.13	0.121
% Waist circumference >80 cm	81.25 (26)	85.71 (42)	0.758
PPWR (kg)	1.2 ± 5.04	0.7 ± 4.97	1.000
PPWR > 0 kg	57.58 (19)	52.94 (27)	0.823
PPWR > 5 kg	15.15 (5)	13.73 (7)	1.000
HbA1c (%)	5.4 ± 0.40	5.4 ± 0.38	0.700
HbA1c (mmol/mol)	35.7 ± 4.38	35.4 ± 4.14	0.700
Fasting LDL-cholesterol (mmol/L)	3.11 ± 0.87	2.92 ± 0.83	0.434
FFQ:			
Total water (g)	2143.8 (1761.4–2437.3)	2211.6 (1804.5–2434.7)	0.791
Total fruit (g)	120.0 (17.2–225.0)	108.3 (51.5–225.0)	0.724
Total vegetables (g)	240.0 (103.0–240.0)	240.0 (120.0–240.0)	0.645
Total meat (g)	99.9 (68.1–137.2)	113.8 (76.2–141.2)	0.426
Total fish (g)	17.5 (9.6–29.6)	15.0 (9.6–23.8)	0.511
Total rest group (g)	149.1 (86.0–466.2)	254.9 (79.7–451.5)	0.448
Daily protein intake (g)	59.8 (54.9–78.9)	64.3 (49.9–74.4)	0.930
Daily fat intake (g)	50.8 (42.1–62.6)	54.3 (44.7–67.1)	0.397
Daily carbohydrate intake (g)	184.6 (150.5–213.9)	184.1 (156.6–226.0)	0.834
Daily fiber intake (g)	18.2 (13.8–20.5)	17.9 (15.0–22.3)	0.867
IPAQ/METs category at time of OGTT			0.813
% Low	13.33 (4)	14.89 (7)	
% Moderate	46.67 (14)	38.30 (18)	
% High	40.00 (12)	46.81 (22)	
STAI-6	12.9 ± 3.44	11.2 ± 2.88	**0.014**
**One year after randomization ^c^**			
% Breastfeeding	81.82 (27)	84.00 (42)	1.000
FPG (mmol/L)	5.37 ± 0.93	5.46 ± 0.64	0.267
Glycemia 60 min (mmol/L)	8.90 ± 2.60	8.69 ± 2.18	0.956
Glycemia 120 min (mmol/L)	7.46 ± 2.75	7.34 ± 1.55	0.475
Timing OGTT (weeks)	66.1 ± 3.84	65.3 ± 3.42	0.387
% T2DM	9.09 (3)	3.92 (2)	1.000
% IFG	25.00 (4)	31.25 (10)	
% IGT	43.75 (7)	40.63 (13)	
% IFG + IGT	31.25 (5)	28.13 (9)	
BMI (kg/m^2^)	26.1 ± 6.03	28.4 ± 6.00	0.084
Mean systolic blood pressure (mmHg)	118.1 ± 15.25	119.7 ± 11.13	0.880
Mean diastolic blood pressure (mmHg)	76.2 ± 9.42	76.6 ± 9.33	0.869
% Hypertension	15.15 (5)	11.76 (6)	0.745
Waist circumference (cm)	86.7 ± 17.55	91.8 ± 13.86	0.057
% Waist circumference >80 cm	59.38 (19)	80.39 (41)	**0.046**
PPWR (kg)	−1.4 ± 6.59	0.6 ± 5.19	0.186
PPWR > 0 kg	42.42 (14)	52.94 (27)	0.379
PPWR > 5 kg	9.09 (3)	17.65 (9)	0.350
HbA1c (%)	5.4 ± 0.54	5.3 ± 0.35	0.703
HbA1c (mmol/mol)	35.5 ± 5.95	34.7 ± 3.77	0.703
Fasting LDL-cholesterol (mmol/L)	2.93 ± 0.87	2.95 ± 0.89	0.967
FFQ:			
Total water (g)	1788.9 (1420.4–2156.7)	2222.2 (1653.4–2564.7)	**0.038**
Total fruit (g)	96.5 (51.5–225.0)	96.5 (51.5–225.0)	0.673
Total vegetables (g)	240.0 (120.0–240.0)	188.6 (120.0–240.0)	0.535
Total meat (g)	82.0 (63.9–135.8)	93.3 (63.9–140.0)	0.654
Total fish (g)	16.6 (10.4–22.5)	13.9 (8.6–22.2)	0.417
Total rest group (g)	119.4 (50.4–500.4)	237.4 (80.0–453.1)	0.469
Daily protein intake (g)	55.7 (42.5–63.4)	55.1 (45.6–66.8)	0.598
Daily fat intake (g)	48.5 (34.4–60.5)	47.4 (37.9–57.2)	0.833
Daily carbohydrate intake (g)	157.7 (131.3–193.1)	169.9 (133.5–208.8)	0.602
Daily fiber intake (g)	15.9 (13.6–19.6)	16.3 (11.7–21.1)	0.876
IPAQ/METs category at time of OGTT			0.341
% Low	10.71 (3)	15.56 (7)	
% Moderate	28.57 (8)	42.22 (19)	
% High	60.71 (17)	42.22 (19)	
% Clinical depression (≥16 on CES-D questionnaire)	30.30 (10)	17.65 (9)	0.193
STAI-6	12.9 ± 3.75	12.5 ± 3.54	0.645
% Recent changes to reduce risk	45.45 (15)	58.82 (30)	0.267
% Planning changes to reduce risk	57.58 (19)	84.31 (43)	**0.010**

^a^ GDM, gestational diabetes mellitus; PCOS, polycystic ovary syndrome; BMI, body mass index; and T2DM, type 2 diabetes mellitus. ^b^ FPG, fasting plasma glucose; OGTT, oral glucose tolerance test; PPWR, postpartum weight retention; LDL-cholesterol, low-density lipoprotein cholesterol; FFQ, Food Frequency Questionnaire; IPAQ, International Physical Activity Questionnaire; METs, metabolic equivalent of task [MET] minutes/week; CES-D, Center for Epidemiologic Studies–Depression; and STAI-6, Spielberger State-Trait Anxiety Inventory. ^c^ IFG, impaired fasting glucose; IGT, impaired glucose tolerance. ^d,e^ Categorical variables were analyzed using Fisher’s exact test and are presented as frequencies % (n); continuous variables were analyzed using the Mann–Whitney U test and are presented as mean ± SD if normally distributed and as median ± IQR if not normally distributed. ^f^ Differences are considered significant at *p*-value < 0.05. Bold means a statistically significant value of *p* < 0.05.

**Table 4:** Diabetes risk perception at one year after randomization: intervention vs. control adjusted for baseline (6–16 weeks postpartum) difference in diabetes risk perception.

Model ^a^	Odds Ratio (95% CI) ^b^	*p*-Value ^c^
Not adjusted for baseline risk perception	0.885 (0.475;1.650)	0.7011
Adjusted for baseline risk perception	0.883 (0.469;1.661)	0.6990

^a^ Adjusted for ‘adequate risk estimation’ on baseline (6–16 weeks postpartum). ^b^ OR, odds ratio; CI, confidence interval. OR >(<) one means higher (lower) probability of overestimating risk in the intervention group vs. control group. ^c^ Differences are considered significant at *p*-value < 0.05.

## Data Availability

All data supporting the findings of this sub-analysis of the Melinda study are available in this article and its Supplementary Materials. Additional data may be provided upon reasonable request to the corresponding author. All data have been anonymized in accordance with ethical and privacy restrictions.

## Supplementary Materials

The following supporting information can be downloaded at https://www.mdpi.com/article/10.3390/jcm14144998/s1. Table S1: Perceived risk of diabetes between participants who stopped early vs. participants with 1 year follow-up; Table S2: Characteristics of intervention group vs. control group of the whole cohort; Table S3: Characteristics of intervention group vs. control group in participants who adequately estimated their diabetes risk at baseline (6–16 weeks postpartum); Table S4: Characteristics of intervention group vs. control group in participants who adequately estimated their diabetes risk at one year post-randomization; Table S5: Intervention group: comparison of groups according to switch (from underestimating to adequately estimating their diabetes risk or vice versa) between baseline and one year post-randomization; and Table S6: Control group: comparison of groups according to switch (from underestimating to adequately estimating their diabetes risk and vice versa) between baseline and one year post-randomization.

## Abbreviations

The following abbreviations are used in this manuscript:

GDM	Gestational Diabetes Mellitus
T2DM	Type 2 Diabetes Mellitus
ACOG	American College of Obstetricians and Gynecologists
ADA	American Diabetes Association
BMI	Body Mass Index
OGTT	Oral Glucose Tolerance Test
FPG	Fasting Plasma Glucose
RCT	Randomized Controlled Trial
WHO	World Health Organization
IFG	Impaired Fasting Glucose
IGT	Impaired Glucose Tolerance
MET	Metabolic Equivalent of Task
IPAQ	International Physical Activity Questionnaire
CES-D	Center for Epidemiologic Studies Depression Scale
FFQ	Food Frequency Questionnaire
STAI-6	State-Trait Anxiety Inventory-6
RPS-DD	Risk Perception Survey for Developing Diabetes
PPWR	Postpartum Weight Retention
HDL	High-Density Lipoprotein
LDL	Low-Density Lipoprotein
PCOS	Polycystic Ovary Syndrome
OR	Odds Ratio
SD	Standard Deviation
IQR	Interquartile Range
CI	Confidence Interval
