# Supplementary material for: Diabetes Risk Perception in Women with a Recent History of Gestational Diabetes Mellitus: A Secondary Analysis from a Belgian Randomized Controlled Trial (MELINDA Study)
Source: J Clin Med. 2025 Jul 15;14(14):4998. doi: 10.3390/jcm14144998 (PMC12294926; doi:10.3390/jcm14144998)
Supplement: Supplementary file 1 [file jcm-14-04998-s001.zip › jcm-3707130-supplementary.pdf]

Supplementary Table S1. Perceived risk of diabetes between participants who stopped early vs. participants with 1 year follow-up

Variable	Early stop (n=73)[a]	1 year follow-up (n=165)[b]	Total (n=238)	P-value[c]
Underestimated their risk at baseline	26.0% (19)	40.6% (67)	36.1% (86)	0.040
Adequately estimated their risk at baseline	74.0% (54)	59.4% (98)	63.9% (152)	

[a]Early stop: refers to 73 participants who only had data available from baseline visit and withdrew from the study early.

[b]1 year follow-up: refers to 165 participants who had data available at both baseline and one year post-randomization.

[c]Differences are considered significant at p-value<0.05. Bold means a statistical significant value of p<0.05.

Supplementary Table S2. Characteristics of intervention group vs. control group of the whole cohort

	Control group (N=84, 50.9%)[d,e]	Intervention group (N=81, 49.1%)[d,e]	p-value[f]
**General characteristics[a]**			
Age (years) baseline	32.8 ± 4.09	31.4 ± 4.15	**0.018**
% Non-Caucasian	20.2 (17)	13.6 (11)	0.302
Highest education<br>%None/primary school<br>%Until age of 15 years<br>%High school<br>%Higher education (bachelor/master)	 1.19 (1)<br>5.95 (5)<br>15.48 (13)<br>77.38 (65)	 0.00 (0)<br>8.64 (7)<br>18.52 (15)<br>72.84 (59)	0.703
% Paid professional activity	83.33 (70)	88.89 (72)	0.371
Monthly net income family<br>%Low income <1500<br>%€1500-5000<br>% >€5000	 6.02 (5)<br>85.54 (71)<br>8.43 (7)	 1.23 (1)<br>88.89 (72)<br>9.88 (8)	0.308
% Living without partner	17.86 (15)	17.28 (14)	1.000
% Currently smoking	7.14 (6)	3.70 (3)	0.496
% Multiparity	55.95 (47)	49.38 (40)	0.437
% History of GDM in previous pregnancy	22.03 (13)	21.28 (10)	1.000
% History of PCOS	3.80 (3)	6.41 (5)	0.495
% History of miscarriage	42.86 (36)	25.93 (21)	**0.033**
Pre-pregnancy BMI (kg/m^2)	27.6 ± 5.64	26.4 ± 5.20	0.154
% First degree family history of T2DM	33.75 (27)	24.68 (19)	0.225
% Second degree family history of T2DM	64.29 (45)	59.15 (42)	0.604
**Baseline (6-16 weeks postpartum)[b]**			
FPG (mmol/L)<br>Glycemia 60 min (mmol/L)<br>Glycemia 120 min (mmol/L)	5.29 ± 0.58<br>9.50 ± 1.87<br>8.10 ± 1.32	5.18 ± 0.58<br>9.45 ± 2.10<br>8.21 ± 1.50	0.185<br>0.748<br>0.577
Timing OGTT (weeks)	12.6 ± 2.52	12.3 ± 3.07	0.109
BMI (kg/ m^2)	27.9 ± 5.67	26.6 ± 5.35	0.111
Mean systolic blood pressure (mmHg)	119.2 ± 12.28	117.2 ± 11.69	0.400
Mean diastolic blood pressure (mmHg)	75.2 ± 10.37	75.1 ± 7.80	0.883
% Hypertension	10.71 (9)	6.17 (5)	0.404
Waist circumference (cm)	93.5 ± 13.43	89.0 ± 12.59	**0.030**
PPWR (kg)	0.9 ± 4.97	0.7 ± 4.62	0.867
PPWR > 0 kg	54.76 (46)	54.32 (44)	0.1000
PPWR > 5kg	14.29 (12)	13.58 (11)	0.1000
HbA1c (%)	5.4 ± 0.39	5.4 ± 0.25	0.511
HbA1c (mmol/mol)	35.5 ± 4.21	35.5 ± 2.77	0.511
Fasting LDL-cholesterol (mmol/L)	6.43 ± 1.81	6.13 ± 1.57	0.302
FFQ:<br>Total water (g)<br>Total fruit (g)<br>Total vegetables (g)<br>Total meat (g)<br>Total fish (g)<br>Total rest group (g)<br>Daily protein intake (g)<br>Daily fat intake (g)<br>Daily carbohydrates intake (g)<br>Daily fiber intake (g)	 2199.1 (1761.4-2437.3)<br>120.0 (51.5-225.0)<br>240.0 (120.0-240.0)<br>108.6 (69.7-141.1)<br>15.0 (9.6-27.5)<br>212.6 (79.7-466.2)<br>63.3 (51.2-74.6)<br>53.1 (42.9-67.1)<br>184.6 (151.0-222.5)<br>18.1 (13.8-21.7)	 2065.0 (1734.3-2498.7)<br>96.5 (51.5-225.0)<br>240.0 (120.0-240.0)<br>115.2 (82.2-150.8)<br>16.6 (8.6-27.5)<br>240.9 (94.3-493.0)<br>61.9 (51.0-77.1)<br>48.8 (41.3-63.3)<br>178.1 (141.0-216.7)<br>18.3 (13.3-21.6)	 0.919<br>0.777<br>0.382<br>0.360<br>0.887<br>0.445<br>0.863<br>0.205<br>0.511<br>0.920
IPAQ/METs category at time of OGTT<br>% Low<br>% Moderate<br>% High	 14.29 (11)<br>41.56 (32)<br>44.16 (34)	 12.50 (10)<br>43.75 (35)<br>43.75 (35)	0.928

STAI-6	11.9 ± 3.20	12.1 ± 3.26	0.598
% Clinical depression (≥16 on CES-D questionnaire)	24.10 (20)	19.75 (16)	0.573
**One year after randomization[c]**			
% Breastfeeding	83.13 (69)	71.60 (58)	0.094
FPG (mmol/L)<br>Glycemia 60 min (mmol/L)<br>Glycemia 120 min (mmol/L)	5.43 ± 0.76<br>8.77 ± 2.34<br>7.39 ± 2.09	5.20 ± 0.51<br>9.00 ± 2.35<br>7.21 ± 2.02	0.061<br>0.338<br>0.803
Timing OGTT (weeks)	65.6 ± 3.59	64.3 ± 3.48	**0.011**
% T2DM<br>% IFG<br>% IGT<br>% IFG + IGT	5.95 (5)<br>29.17 (14)<br>41.67 (20)<br>29.17 (14)	3.70 (3)<br>23.26 (10)<br>51.16 (22)<br>25.58 (11)	0.662
BMI (kg/ m^2)	27.7 ± 6.08	25.7 ± 5.48	0.054
Mean systolic blood pressure (mmHg)	119.1 ± 12.84	118.5 ± 12.71	0.779
Mean diastolic blood pressure (mmHg)	76.4 ± 9.31	77.4 ± 11.29	0.452
% Hypertension	13.10 (11)	16.05 (13)	0.662
Waist circumference (cm)	89.8 ± 15.49	85.4 ± 13.31	0.052
PPWR (kg)	-0.17 ± 5.828	-1.60 ± 5.294	0.135
PPWR > 0 kg	48.81 (41)	39.51 (32)	0.273
PPWR > 5kg	14.29 (12)	7.41 (6)	0.213
HbA1c (%)	5.4 ± 0.43	5.3 ± 0.26	0.999
HbA1c (mmol/mol)	35.0 ± 4.69	34.6 ± 2.82	0.999
Fasting LDL-cholesterol (mmol/L)	6.31 ± 1.88	5.99 ± 1.72	0.160
FFQ:<br>Total water (g)<br>Total fruit (g)<br>Total vegetables (g)<br>Total meat (g)<br>Total fish (g)<br>Total rest group (g)<br>Daily protein intake (g)<br>Daily fat intake (g)<br>Daily carbohydrates intake (g)<br>Daily fiber intake (g)	 1973.0 (1520.4-2415.7)<br>96.5 (51.5-225.0)<br>240.0 (120.0-240.0)<br>92.2 (63.9-140.0)<br>15.0 (9.6-22.2)<br>219.8 (63.5-466.9)<br>55.6 (43.8-66.3)<br>48.0 (37.7-57.7)<br>162.9 (132.4-200.6)<br>16.0 (12.3-20.6)	 1933.4 (1570.7-2300.0)<br>96.5 (51.5-225.0)<br>240.0 (120.0-240.0)<br>90.7 (69.7-133.1)<br>16.6 (9.6-23.8)<br>197.1 (60.4-304.4)<br>54.7 (44.1-68.4)<br>43.2 (34.7-56.2)<br>146.1 (124.4-178.2)<br>15.9 (12.4-18.8)	 0.610<br>0.659<br>0.774<br>0.822<br>0.307<br>0.225<br>0.746<br>0.265<br>0.074<br>0.602
IPAQ/METs category at time of OGTT<br>% Low<br>% Moderate<br>% High	 13.70 (10)<br>36.99 (27)<br>49.32 (36)	 1.39 (1)<br>41.67 (30)<br>56.94 (41)	**0.019**
STAI-6	12.7 ± 3.60	12.4 ± 3.71	0.492
% Clinical depression (≥16 on CES-D questionnaire)	22.62 (19)	30.86 (25)	0.291
% Recent changes to reduce risk	53.57 (45)	49.38 (40)	0.642
% Planning changes to reduce risk	73.81 (62)	81.48 (66)	0.266

[a]GDM, gestational diabetes mellitus; PCOS, polycystic ovary syndrome; BMI, body mass index; T2DM, type 2 diabetes mellitus.

[b]FPG, fasting plasma glucose; OGTT, oral glucose tolerance test; PPWR, postpartum weight retention; LDL-cholesterol, low density lipoprotein cholesterol; FFQ, Food Frequency Questionnaire; IPAQ, International Physical Activity Questionnaire; METs, metabolic equivalent of task [MET] minutes/week; CES-D, Center for Epidemiologic Studies–Depression; STAI-6, Spielberger State-Trait Anxiety Inventory.

[c]IFG, impaired fasting glucose; IGT, impaired glucose tolerance.

[d, e]Categorical variables are presented as frequencies %(n); continuous variables are presented as mean ± SD if normally distributed and as median ± IQR if not normally distributed.

[f]Differences are considered significant at p-value<0.05. Bold means a statistical significant value of p<0.05.

Supplementary Table S3. Characteristics of intervention group vs. control group in participants who adequately estimated their diabetes risk at baseline (6-16 weeks postpartum)

	Control group (N=55, 57.3%)[d,e]	Intervention group (N=41, 42.7%)[d,e]	p-value[f]
**General characteristics[a]**			
Age (years)	32.9 ± 3.67	31.5 ± 4.23	0.067
% Non-Caucasian	16.36 (9)	12.20 (5)	0.771
Highest education			0.834
%None/primary school	0.00 (0)	0.00 (0)	
%Until age of 15 years	7.27 (4)	9.76 (4)	
%High school	20.00 (11)	17.07 (7)	
%Higher education (bachelor/master)	72.73 (40)	73.17 (30)	
% Paid professional activity	81.82 (45)	92.68 (38)	0.145
Monthly net income family			0.339
%Low income <1500	3.64 (2)	0.00 (0)	
%€1500-5000	90.91 (50)	87.80 (36)	
% >€5000	5.45 (3)	12.20 (5)	
% Living without partner	20.00 (11)	19.51 (8)	1.000
% Currently smoking	10.91 (6)	2.44 (1)	0.233
% Multiparity	50.91 (28)	56.10 (23)	0.682
% History of GDM in previous pregnancy	18.92 (7)	22.22 (6)	0.763
% History of PCOS	1.92 (1)	5.00 (2)	0.578
% History of miscarriage	38.18 (21)	31.71 (13)	0.527
Pre-pregnancy BMI (kg/m^2)	28.2 ± 5.59	27.3 ± 5.47	0.326
% First degree family history of T2DM	40.74 (22)	30.77 (12)	0.386
% Second degree family history of T2DM	73.33 (33)	75.00 (27)	1.000
**Baseline (6-16 weeks postpartum)[b]**			
FPG (mmol/L)	5.33 ± 0.59	5.28 ± 0.54	0.529
Glycemia 60 min (mmol/L)	9.51 ± 1.92	9.34 ± 2.16	0.517
Glycemia 120 min (mmol/L)	8.18 ± 1.24	8.01 ± 1.49	0.591
Timing OGTT (weeks)	12.6 ± 2.30	11.7 ± 1.94	**0.022**
BMI (kg/ m^2)	28.5 ± 5.49	27.8 ± 5.68	0.364
Mean systolic blood pressure (mmHg)	118.8 ± 11.45	120.3 ± 10.10	0.467
Mean diastolic blood pressure (mmHg)	75.3 ± 9.08	76.4 ± 8.08	0.550
% Hypertension	9.09 (5)	7.32 (3)	1.000
Waist circumference (cm)	94.9 ± 13.35	91.8 ± 12.71	0.182
% Waist circumference >80 cm	86.79 (46)	80.49 (33)	0.571
PPWR (kg)	0.8 ± 5.24	1.3 ± 4.65	0.591
PPWR > 0 kg	56.36 (31)	56.10 (23)	1.000
PPWR > 5kg	14.55 (8)	17.07 (7)	0.781
HbA1c (%)	5.4 ± 0.41	5.5 ± 0.19	0.164
HbA1c (mmol/mol)	35.5 ± 4.45	36.1 ± 2.06	0.164
Fasting LDL-cholesterol (mmol/L)	3.06 ± 0.88	2.97 ± 0.67	0.663
FFQ:			
Total water (g)	2213.0 (1821.0-2437.3)	2038.3 (1734.3-2498.7)	0.539
Total fruit (g)	120.0 (51.5-225.0)	96.5 (51.5-176.9)	0.343
Total vegetables (g)	188.6 (120.0-240.0)	240.0 (120.0-240.0)	0.688
Total meat (g)	113.3 (76.2-141.1)	127.7 (90.3-167.5)	0.284
Total fish (g)	15.0 (8.6-22.2)	16.6 (8.6-27.5)	0.481
Total rest group (g)	212.6 (86.0-451.5)	378.6 (122.6-661.4)	0.084
Daily protein intake (g)	61.9 (52.1-77.0)	64.9 (52.3-80.0)	0.604
Daily fat intake (g)	52.2 (42.1-67.1)	54.5 (44.4-65.0)	0.965
Daily carbohydrates intake (g)	180.4 (146.9-213.6)	188.0 (149.8-230.5)	0.459
Daily fiber intake (g)	18.1 (12.8-21.9)	17.8 (13.3-20.7)	0.970
IPAQ/METs category at time of OGTT			0.959

% Low<br>% Moderate<br>% High	15.38 (8)<br>42.31 (22)<br>42.31 (22)	12.50 (5)<br>45.00 (18)<br>42.50 (17)	
STAI-6	12.4 ± 3.17	12.6 ± 3.13	0.458
**One year after randomization[c]**			
% Breastfeeding	79.76 (67)	67.90 (55)	0.110
FPG (mmol/L)<br>Glycemia 60 min (mmol/L)<br>Glycemia 120 min (mmol/L)	5.39 ± 0.80<br>8.88 ± 2.46<br>7.58 ± 2.33	5.36 ± 0.46<br>9.00 ± 1.99<br>7.13 ± 2.04	0.728<br>0.539<br>0.493
Timing OGTT (weeks)	65.8 ± 3.38	63.8 ± 2.90	**0.004**
% T2DM<br>% IFG<br>% IGT<br>% IFG + IGT	5.45 (3)<br>19.35 (6)<br>45.16 (14)<br>35.48 (11)	2.44 (1)<br>29.17 (7)<br>45.83 (11)<br>25.00 (6)	0.633<br>0.648<br>0.648<br>0.648
BMI (kg/ m^2)	27.9 ± 5.94	27.0 ± 5.98	0.341
Mean systolic blood pressure (mmHg)	119.6 ± 14.21	122.7 ± 12.20	0.271
Mean diastolic blood pressure (mmHg)	77.3 ± 9.15	80.7 ± 9.69	0.120
% Hypertension	12.73 (7)	19.51 (8)	0.405
Waist circumference (cm)	90.9 ± 15.95	88.1 ± 13.66	0.312
% Waist circumference >80 cm	74.55 (41)	68.29 (28)	0.647
PPWR (kg)	-0.8 ± 5.03	-0.8 ± 5.14	0.929
PPWR > 0 kg	45.45 (25)	41.46 (17)	0.835
PPWR > 5kg	10.91 (6)	9.76 (4)	0.954
HbA1c (%)	5.4 ± 0.46	5.4 ± 0.27	0.484
HbA1c (mmol/mol)	35.3 ± 5.06	35.2 ± 2.93	0.484
Fasting LDL-cholesterol (mmol/L)	2.89 ± 0.82	2.91 ± 0.85	0.818
FFQ:<br>Total water (g)<br>Total fruit (g)<br>Total vegetables (g)<br>Total meat (g)<br>Total fish (g)<br>Total rest group (g)<br>Daily protein intake (g)<br>Daily fat intake (g)<br>Daily carbohydrates intake (g)<br>Daily fiber intake (g)	 1914.5 (1484.8-2551.5)<br>96.5 (51.5-225.0)<br>188.6 (120.0-240.0)<br>91.1 (63.9-145.8)<br>12.0 (8.6-18.5)<br>220.9 (80.0-472.0)<br>55.1 (43.0-66.1)<br>50.7 (37.5-57.8)<br>157.7 (117.4-208.7)<br>15.8 (12.6-19.7)	 1696.4 (1492.0-2300.0)<br>96.5 (32.2-225.0)<br>240.0 (120.0-240.0)<br>102.3 (75.0-145.8)<br>16.6 (10.4-27.5)<br>212.7 (77.4-434.3)<br>58.7 (51.1-68.9)<br>48.6 (38.9-58.2)<br>151.0 (127.3-183.4)<br>15.6 (12.2-18.2)	 0.270<br>0.354<br>0.915<br>0.276<br>0.087<br>0.731<br>0.454<br>0.873<br>0.461<br>0.581
IPAQ/METs category at time of OGTT<br>% Low<br>% Moderate<br>% High	 10.42 (5)<br>37.50 (18)<br>52.08 (25)	 2.78 (1)<br>41.67 (15)<br>55.56 (20)	0.488
% Clinical depression<br>(≥16 on CES-D questionnaire)	25.45 (14)	29.27 (12)	0.817
STAI-6	13.0 ± 3.61	12.4 ± 3.81	0.300
% Recent changes to reduce risk	54.55 (30)	51.22 (21)	0.837
% Planning changes to reduce risk	70.91 (39)	80.49 (33)	0.345

[a]GDM, gestational diabetes mellitus; PCOS, polycystic ovary syndrome; BMI, body mass index; T2DM, type 2 diabetes mellitus.

[b]FPG, fasting plasma glucose; OGTT, oral glucose tolerance test; PPWR, postpartum weight retention; LDL-cholesterol, low density lipoprotein cholesterol; FFQ, Food Frequency Questionnaire; IPAQ, International Physical Activity Questionnaire; METs, metabolic equivalent of task [MET] minutes/week; CES-D, Center for Epidemiologic Studies–Depression; STAI-6, Spielberger State-Trait Anxiety Inventory.

[c]IFG, impaired fasting glucose; IGT, impaired glucose tolerance.

[d, e]Categorical variables are presented as frequencies %(n); continuous variables are presented as mean ± SD if normally distributed and as median ± IQR if not normally distributed.

[f]Differences are considered significant at p-value<0.05. Bold means a statistical significant value of p<0.05.

Supplementary Table S4. Characteristics of intervention group vs. control group in participants who adequately estimated their diabetes risk at one year post-randomization

	Control group (N=49, 51.6%)[d,e]	Intervention group (N=46, 48.4%)[d,e]	p-value[f]
**General characteristics[a]**			
Age (years)	32.2 ± 3.83	31.8 ± 3.79	0.429
% Non-Caucasian	19.61 (10)	10.87 (5)	0.272
Highest education			0.197
%None/primary school	1.96 (1)	0.00 (0)	
%Until age of 15 years	7.84 (4)	0.00 (0)	
%High school	13.73 (7)	17.39 (8)	
%Higher education (bachelor/master)	76.47 (39)	82.61 (38)	
% Paid professional activity	88.24 (45)	82.61 (42)	0.744
Monthly net income family			0.276
%Low income <1500	6.00 (3)	0.00 (0)	
%€1500-5000	84.00 (42)	84.78 (39)	
% >€5000	10.00 (5)	15.22 (7)	
% Living without partner	17.65 (9)	8.70 (4)	0.242
% Currently smoking	3.92 (2)	0.0 (0)	0.496
% Multiparity	60.78 (31)	47.83 (22)	0.225
% History of GDM in previous pregnancy	21.05 (8)	18.52 (5)	1.000
% History of PCOS	4.17 (2)	4.44 (2)	1.000
% History of miscarriage	43.14 (22)	26.09 (12)	0.092
Pre-pregnancy BMI (kg/m^2)	28.1 ± 5.53	25.3 ± 5.19	**0.007**
% First degree family history of T2DM	31.25 (15)	25.00 (11)	0.644
% Second degree family history of T2DM	68.29 (28)	62.79 (27)	0.651
**Baseline (6-16 weeks postpartum)[b]**			
FPG (mmol/L)	5.37 ± 0.58	5.16 ± 0.58	0.100
Glycemia 60 min (mmol/L)	9.32 ± 1.82	9.12 ± 2.17	0.731
Glycemia 120 min (mmol/L)	7.83 ± 1.44	8.13 ± 1.26	0.390
Timing OGTT (weeks)	12.9 ± 2.52	12.4 ± 2.89	0.114
BMI (kg/ m^2)	28.4 ± 5.66	25.8 ± 5.24	**0.011**
Mean systolic blood pressure (mmHg)	119.1 ± 12.61	117.5 ± 10.53	0.641
Mean diastolic blood pressure (mmHg)	76.3 ± 10.07	74.9 ± 7.21	0.397
% Hypertension	9.80 (5)	4.35 (2)	0.440
Waist circumference (cm)	95.2 ± 14.13	87.6 ± 12.04	**0.010**
% Waist circumference >80 cm	85.71 (42)	71.74 (33)	0.131
PPWR (kg)	0.7 ± 4.97	1.3 ± 3.88	0.649
PPWR > 0 kg	52.94 (27)	60.87 (28)	0.539
PPWR > 5kg	13.73 (7)	10.87 (5)	0.763
HbA1c (%)	5.4 ± 0.38	5.4 ± 0.22	0.320
HbA1c (mmol/mol)	35.4 ± 4.14	35.6 ± 2.38	0.320
Fasting LDL-cholesterol (mmol/L)	2.92 ± 0.83	2.89 ± 0.71	0.835
FFQ:			
Total water (g)	2211.6 (1804.5-2434.7)	2042.0 (1653.8-2498.7)	0.580
Total fruit (g)	108.3 (51.5-225.0)	96.5 (51.5-225.0)	0.809
Total vegetables (g)	240.0 (120.0-240.0)	240.0 (120.0-240.0)	0.813
Total meat (g)	113.8 (76.2-141.2)	115.2 (82.2-150.8)	0.676
Total fish (g)	15.0 (9.6-23.8)	18.5 (8.6-29.7)	0.318
Total rest group (g)	254.9 (79.7-451.5)	262.8 (92.8-493.0)	0.783
Daily protein intake (g)	64.3 (49.9-74.4)	60.5 (49.9-77.8)	0.466
Daily fat intake (g)	54.3 (44.7-67.1)	48.7 (42.3-57.9)	0.075
Daily carbohydrates intake (g)	184.1 (156.6-226.0)	171.6 (140.0-216.7)	0.331
Daily fiber intake (g)	17.9 (15.0-22.3)	18.3 (11.4-21.5)	0.555
IPAQ/METs category at time of OGTT			0.887

% Low<br>% Moderate<br>% High	14.89 (7)<br>38.30 (18)<br>46.81 (22)	17.39 (8)<br>41.30 (19)<br>41.30 (19)	
STAI-6	11.2 ± 2.88	11.9 ± 3.01	0.187
**One year after randomization[c]**			
% Breastfeeding	82.35 (42)	71.74 (33)	0.234
FPG (mmol/L)<br>Glycemia 60 min (mmol/L)<br>Glycemia 120 min (mmol/L)	5.46 ± 0.64<br>8.69 ± 2.18<br>7.34 ± 1.55	5.22 ± 0.50<br>9.06 ± 2.29<br>7.14 ± 2.17	0.064<br>0.458<br>0.532
Timing OGTT (weeks)	65.3 ± 3.42	64.4 ± 3.33	0.245
% T2DM<br>% IFG<br>% IGT<br>% IFG + IGT	3.92 (2)<br>31.25 (10)<br>40.63 (13)<br>28.13 (9)	4.35 (2)<br>26.92 (7)<br>50.00 (13)<br>23.08 (6)	0.838
BMI (kg/ m^2)	28.4 ± 6.00	25.1 ± 5.58	**0.005**
Mean systolic blood pressure (mmHg)	119.7 ± 11.13	117.6 ± 11.30	0.544
Mean diastolic blood pressure (mmHg)	76.6 ± 9.33	78.4 ± 9.89	0.408
% Hypertension	11.76 (6)	15.22 (7)	0.767
Waist circumference (cm)	91.8 ± 13.86	84.1 ± 13.54	**0.005**
% Waist circumference >80 cm	80.39 (41)	58.70 (27)	**0.026**
PPWR (kg)	0.6 ± 5.19	-0.4 ± 4.43	0.302
PPWR > 0 kg	52.94 (27)	43.48 (20)	0.418
PPWR > 5kg	17.65 (9)	8.70 (4)	0.242
HbA1c (%)	5.3 ± 0.35	5.3 ± 0.25	0.928
HbA1c (mmol/mol)	34.7 ± 3.77	34.6 ± 2.76	0.928
Fasting LDL-cholesterol (mmol/L)	2.95 ± 0.89	2.88 ± 0.80	0.680
FFQ:<br>Total water (g)<br>Total fruit (g)<br>Total vegetables (g)<br>Total meat (g)<br>Total fish (g)<br>Total rest group (g)<br>Daily protein intake (g)<br>Daily fat intake (g)<br>Daily carbohydrates intake (g)<br>Daily fiber intake (g)	 2222.2 (1653.4-2564.7)<br>96.5 (51.5-225.0)<br>188.6 (120.0-240.0)<br>93.3 (63.9-140.0)<br>13.9 (8.6-22.2)<br>237.4 (80.0-453.1)<br>55.1 (45.6-66.8)<br>47.4 (37.9-57.2)<br>169.9 (133.5-208.8)<br>16.3 (11.7-21.1)	 1828.5 (1568.8-2318.63)<br>95.4 (51.5-225.0)<br>240.0 (120.0-240.0)<br>88.8 (69.7-127.7)<br>16.6 (10.8-27.53)<br>198.0 (56.9-304.4)<br>54.5 (44.1-64.5)<br>43.7 (36.8-55.1)<br>144.6 (120.5-178.2)<br>16.0 (13.5-18.6)	 0.096<br>0.236<br>0.494<br>0.571<br>0.076<br>0.161<br>0.499<br>0.378<br>0.077<br>0.696
IPAQ/METs category at time of OGTT<br>% Low<br>% Moderate<br>% High	 15.56 (7)<br>42.22 (19)<br>42.22 (19)	 2.33 (1)<br>44.19 (19)<br>53.49 (23)	0.099
% Clinical depression (≥16 on CES-D questionnaire)	17.65 (9)	30.43 (14)	0.158
STAI-6	12.5 ± 3.54	12.3 ± 3.63	0.513
% Recent changes to reduce risk	58.82 (30)	39.13 (18)	0.068
% Planning changes to reduce risk	84.31 (43)	84.78 (39)	1.000

[a]GDM, gestational diabetes mellitus; PCOS, polycystic ovary syndrome; BMI, body mass index; T2DM, type 2 diabetes mellitus.

[b]FPG, fasting plasma glucose; OGTT, oral glucose tolerance test; PPWR, postpartum weight retention; LDL-cholesterol, low density lipoprotein cholesterol; FFQ, Food Frequency Questionnaire; IPAQ, International Physical Activity Questionnaire; METs, metabolic equivalent of task [MET] minutes/week; CES-D, Center for Epidemiologic Studies–Depression; STAI-6, Spielberger State-Trait Anxiety Inventory.

[c]IFG, impaired fasting glucose; IGT, impaired glucose tolerance.

[d, e]Categorical variables are presented as frequencies %(n); continuous variables are presented as mean ± SD if normally distributed and as median ± IQR if not normally distributed.

[f]Differences are considered significant at p-value<0.05. Bold means a statistical significant value of p<0.05.

Supplementary Table S5. Intervention group: comparison of groups according to switch (from underestimating to adequately estimating their diabetes risk or vice versa) between baseline and one year post-randomization

	U/U (N=21, 25.9%)[d,h, l, j]	U/A (N=19, 23.5%)[e, h, l, j, k]	A/U (N=14, 17.3%)[f, h, l, j, k]	A/A (N=27, 33.3%)[g, h, l, k]	**Pairwise comparisons**						
					**p-value[l]**	**1 vs 2[l]**	**1 vs 3[l]**	**1 vs 4[l]**	**2 vs 3[l]**	**2 vs 4[l]**	**3 vs 4[l]**
**General characteristics[a]**											
Age (years)	30.1 ± 4.23	32.4 ± 3.75	31.6 ± 5.06	31.4 ± 3.83	0.297	0.057	0.279	0.353	0.701	0.287	0.709
% Non-Caucasian	19.05 (4)	10.53 (2)	14.29 (2)	11.11 (3)	0.889	0.664	1.000	0.683	1.000	1.000	1.000
Highest education					**0.042**	0.323	0.510	0.136	**0.046**	0.700	**0.009**
%None/primary school	0.00 (0)	0.00 (0)	0.00 (0)	0.00 (0)							
%Until age of 15 years	14.29 (3)	0.00 (0)	28.57 (4)	0.00 (0)							
%High school	19.05 (4)	21.05 (4)	21.43 (3)	14.81 (4)							
%Higher education (bachelor/master)	66.67 (14)	78.95 (15)	50.00 (7)	85.19 (23)							
% Paid professional activity	80.95 (17)	89.47 (17)	92.86 (13)	92.59 (25)	0.668	0.664	0.627	0.383	1.000	1.000	1.000
Monthly net income family					0.254	0.098	0.647	0.074	0.620	1.000	0.645
%Low income <1500	4.76 (1)	0.00 (0)	0.00 (0)	0.00 (0)							
%€1500-5000	95.24 (20)	84.21 (16)	92.86 (13)	85.19 (23)							
% >€5000	0.00 (0)	15.79 (3)	7.14 (1)	14.81 (4)							
% Living without partner	23.81 (5)	5.26 (1)	35.71 (5)	11.11 (3)	0.092	0.186	0.474	0.272	0.062	0.632	0.097
% Currently smoking	9.52 (2)	0.00 (0)	7.14 (1)	0.00 (0)	0.199	0.488	1.000	0.186	0.424	-	0.341
% Multiparity	38.10 (8)	47.37 (9)	71.43 (10)	48.15 (13)	0.279	0.554	0.053	0.486	0.167	0.958	0.154
% History of GDM in previous pregnancy	20.00 (2)	20.00 (2)	30.00 (3)	17.65 (3)	0.895	1.000	0.606	0.879	0.606	0.879	0.456
% History of PCOS	15.00 (3)	0.00 (0)	0.00 (0)	7.41 (2)	0.201	0.087	0.143	0.404	-	0.238	0.314
% History of miscarriage	28.57 (6)	10.53 (2)	21.43 (3)	37.04 (10)	0.230	0.154	0.636	0.537	0.388	**0.044**	0.308
Pre-pregnancy BMI (kg/m²)	26.9 ± 4.84	23.8 ± 4.28	29.0 ± 5.00	26.4 ± 5.56	**0.032**	**0.047**	0.195	0.582	**0.007**	0.157	0.076
% First degree family history of T2DM	20.00 (4)	16.67 (3)	30.77 (4)	30.77 (8)	0.651	0.791	0.481	0.410	0.354	0.288	1.000
% Second degree family history of T2DM	38.89 (7)	47.06 (8)	80.00 (8)	73.08 (19)	**0.047**	0.625	**0.037**	**0.023**	0.093	0.084	0.667
**Baseline (6-16 weeks postpartum)[b]**											
FPG (mmol/L)	5.14 ± 0.61	5.00 ± 0.60	5.29 ± 0.56	5.27 ± 0.55	0.492	0.551	0.590	0.603	0.215	0.137	1.000
Glycemia 60 min (mmol/L)	10.06 ± 1.87	9.01 ± 2.14	9.61 ± 2.07	9.20 ± 2.23	0.172	0.053	0.613	0.069	0.343	0.729	0.309
Glycemia 120 min (mmol/L)	8.55 ± 1.73	8.29 ± 1.23	7.99 ± 1.89	8.01 ± 1.29	0.502	0.310	0.363	0.134	0.956	0.746	0.815
Timing OGTT (weeks)	12.4 ± 4.02	13.4 ± 3.69	11.7 ± 1.95	11.6 ± 1.97	0.284	0.195	0.919	0.860	0.106	0.069	0.731
BMI (kg/ m²)	26.3 ± 5.18	24.4 ± 4.17	29.8 ± 5.15	26.7 ± 5.75	**0.041**	0.244	0.077	0.852	**0.005**	0.181	0.067

Mean systolic blood pressure (mmHg)	112.6 ± 11.28	115.4 ± 13.75	123.0 ± 13.81	118.9 ± 7.46	0.062	0.597	**0.030**	**0.039**	0.126	0.237	0.191
Mean diastolic blood pressure (mmHg)	74.4 ± 7.33	72.9 ± 7.52	76.6 ± 10.43	76.3 ± 6.79	0.166	0.315	0.381	0.204	0.139	**0.044**	0.890
% Hypertension	0.00 (0)	10.53 (2)	21.43 (3)	0.00 (0)	**0.024**	0.127	**0.027**	-	0.388	0.085	**0.012**
Waist circumference (cm)	87.7 ± 10.62	84.5 ± 13.36	95.6 ± 15.61	89.8 ± 10.72	0.164	0.378	0.138	0.499	0.058	0.147	0.232
% Waist circumference >80 cm	80.95 (17)	63.16 (12)	85.71 (12)	77.78 (21)	0.423	0.208	0.714	0.788	0.150	0.278	0.543
PPWR (kg)	-1.5 ± 5.48	1.7 ± 2.42	2.1 ± 4.70	1.0 ± 4.67	**0.025**	**0.008**	**0.040**	**0.046**	0.316	0.211	0.284
PPWR > 0 kg	28.57 (6)	78.95 (15)	71.43 (10)	48.15 (13)	**0.006**	**0.001**	**0.013**	0.169	0.618	**0.035**	0.154
PPWR > 5kg	14.29 (3)	5.26 (1)	21.43 (3)	14.81 (4)	0.594	0.342	0.583	0.959	0.160	0.305	0.594
HbA1c (%)	5.3 ± 0.32	5.4 ± 0.26	5.5 ± 0.20	5.4 ± 0.18	0.071	0.413	**0.023**	0.072	0.113	0.477	0.131
HbA1c (mmol/mol)	34.4 ± 3.50	35.3 ± 2.88	36.9 ± 2.15	35.8 ± 1.96	0.071	0.413	**0.023**	0.072	0.113	0.477	0.131
Fasting LDL-cholesterol (mmol/L)	2.59 ± 0.72	2.92 ± 0.82	3.15 ± 0.74	2.87 ± 0.63	0.072	0.136	**0.023**	0.064	0.236	0.936	0.182
FFQ:											
Total water (g)	2366.2 (1945.3-2507.3)	2002.9 (1606.6-2433.0)	1973.2 (1505.1-2453.1)	2067.0 (1734.3-2534.2)	0.515	0.184	0.274	0.360	0.899	0.503	0.690
Total fruit (g)	120.0 (51.5-225.0)	96.5 (51.5-225.0)	51.5 (17.2-176.9)	96.5 (51.5-176.9)	0.367	0.889	0.123	0.397	0.181	0.592	0.236
Total vegetables (g)	240.0 (240.0-283.0)	188.6 (94.3-240.0)	214.3 (120.0-360.0)	240.0 (120.0-240.0)	0.495	0.306	0.875	0.138	0.424	0.835	0.431
Total meat (g)	98.2 (64.2-136.7)	118.5 (84.4-149.0)	132.8 (108.6-192.6)	114.3 (69.7-156.4)	0.272	0.448	0.055	0.513	0.196	1.000	0.169
Total fish (g)	10.8 (8.6-25.6)	18.5 (7.8-38.3)	13.1 (7.0-38.4)	18.5 (9.2-27.5)	0.718	0.401	0.946	0.473	0.455	0.815	0.386
Total rest group (g)	213.7 (85.0-326.9)	132.0 (57.5-429.0)	476.4 (195.5-666.8)	374.0 (114.8-532.7)	0.096	0.892	**0.038**	0.124	0.061	0.174	0.372
Daily protein intake (g)	56.2 (50.6-72.1)	60.9 (49.9-70.2)	71.0 (65.6-84.4)	60.0 (49.8-80.0)	0.189	0.935	0.089	0.852	**0.033**	0.929	0.081
Daily fat intake (g)	42.7 (37.6-61.5)	46.5 (43.1-57.8)	64.5 (46.2-81.5)	50.0 (41.6-63.2)	0.063	0.432	**0.021**	0.430	**0.028**	0.841	**0.038**
Daily carbohydrates intake (g)	179.2 (141.0-210.1)	166.5 (137.9-189.6)	204.8 (173.9-233.4)	174.0 (143.7-223.1)	0.307	0.291	0.219	0.819	0.056	0.372	0.402
Daily fiber intake (g)	19.3 (15.5-23.5)	18.3 (12.0-21.6)	17.5 (16.3-20.7)	17.9 (11.1-20.7)	0.684	0.401	0.880	0.309	0.623	0.704	0.450
IPAQ/METs category at time of OGTT					0.340	0.052	0.227	0.380	0.812	0.491	1.000
% Low	0.00 (0)	26.32 (5)	15.38 (2)	11.11 (3)							
% Moderate	47.62 (10)	36.84 (7)	46.15 (6)	44.44 (12)							
% High	52.38 (11)	36.84 (7)	38.46 (5)	44.44 (12)							
STAI-6	11.7 ± 3.47	11.3 ± 3.25	13.1 ± 3.72	12.3 ± 2.81	0.326	0.634	0.204	0.451	0.142	0.188	0.390
**One year after randomization[c]**											
% Breastfeeding	76.19 (16)	84.21 (16)	42.86 (6)	62.96 (17)	0.069	0.698	0.075	0.366	**0.024**	0.184	0.322
FPG (mmol/L)	5.03 ± 0.44	5.06 ± 0.59	5.41 ± 0.58	5.33 ± 0.39	**0.049**	0.914	0.105	**0.024**	0.108	**0.037**	0.923
Glycemia 60 min (mmol/L)	8.87 ± 2.89	9.14 ± 2.51	9.01 ± 1.73	9.00 ± 2.16	0.991	0.871	0.960	0.669	0.985	0.982	0.989
Glycemia 120 min (mmol/L)	7.25 ± 2.03	7.34 ± 2.08	7.37 ± 1.55	7.00 ± 2.27	0.848	0.684	0.699	0.560	0.985	0.562	0.474
Timing OGTT (weeks)	64.0 ± 3.94	65.6 ± 3.92	64.1 ± 3.46	63.6 ± 2.61	0.292	0.124	0.609	0.706	0.288	0.075	0.824
% T2DM	4.76 (1)	5.26 (1)	0.00 (0)	3.70 (1)	0.865	0.942	0.407	0.856	0.383	0.798	0.466
% IFG	12.50 (1)	18.18 (2)	22.22 (2)	33.33 (5)	0.852	0.638	0.745	0.552	0.935	0.485	0.748
% IGT	50.00 (4)	63.64 (7)	55.56 (5)	40.00 (6)	0.852	0.638	0.745	0.552	0.935	0.485	0.748
% IFG + IGT	37.50 (3)	18.18 (2)	22.22 (2)	26.67 (4)	0.852	0.638	0.745	0.552	0.935	0.485	0.748
BMI (kg/ m²)	25.3 ± 4.83	23.5 ± 4.35	28.4 ± 5.59	26.2 ± 6.14	0.077	0.203	0.077	0.670	**0.021**	0.129	0.174

Mean systolic blood pressure (mmHg)	115.8 ± 13.33	112.7 ± 10.26	125.9 ± 14.27	121.0 ± 10.88	**0.030**	0.524	0.059	0.148	**0.013**	**0.026**	0.350
Mean diastolic blood pressure (mmHg)	73.7 ± 14.20	74.3 ± 9.08	79.6 ± 10.21	81.3 ± 9.55	0.096	0.903	0.281	0.072	0.126	**0.025**	0.772
% Hypertension	14.29 (3)	10.53 (2)	21.43 (3)	18.52 (5)	0.825	0.720	0.583	0.696	0.388	0.457	0.824
Waist circumference (cm)	84.2 ± 11.99	80.9 ± 13.20	91.5 ± 13.66	86.3 ± 13.58	0.181	0.432	0.099	0.732	0.053	0.184	0.248
% Waist circumference >80 cm	42.86 (9)	52.63 (10)	78.57 (11)	62.96 (17)	0.179	0.536	**0.036**	0.165	0.126	0.483	0.308
PPWR (kg)	-4.2 ± 6.08	-0.5 ± 3.75	-1.6 ± 5.64	-0.4 ± 4.92	0.170	0.062	0.189	**0.045**	1.000	0.929	0.731
PPWR > 0 kg	28.57 (6)	47.37 (9)	42.86 (6)	40.74 (11)	0.654	0.220	0.383	0.382	0.797	0.655	0.896
PPWR > 5kg	4.76 (1)	5.26 (1)	7.14 (1)	11.11 (3)	0.830	0.942	0.766	0.430	0.823	0.488	0.685
HbA1c (%)	5.2 ± 0.22	5.3 ± 0.26	5.4 ± 0.30	5.4 ± 0.25	0.216	0.953	0.147	0.107	0.186	0.160	0.681
HbA1c (mmol/mol)	33.8 ± 2.44	34.1 ± 2.80	35.6 ± 3.33	35.0 ± 2.72	0.216	0.953	0.147	0.107	0.186	0.160	0.681
Fasting LDL-cholesterol (mmol/L)	2.57 ± 0.82	2.81 ± 0.64	2.88 ± 0.76	2.92 ± 0.91	0.260	0.132	0.092	0.110	0.955	0.863	0.838
FFQ:											
Total water (g)	2047.6 (1782.0-2221.9)	2262.8 (1666.8-2318.6)	1724.6 (1569.5-2300.0)	1696.4 (1464.8-2323.9)	0.522	0.705	0.372	0.212	0.412	0.305	0.794
Total fruit (g)	120.0 (94.3-225.0)	94.3 (51.5-176.9)	74.0 (32.2-225.0)	96.5 (51.5-225.0)	0.356	0.068	0.300	0.219	1.000	0.564	0.845
Total vegetables (g)	240.0 (120.0-240.0)	240.0 (120.0-240.0)	180.0 (120.0-240.0)	240.0 (120.0-240.0)	0.859	0.819	0.584	0.861	0.409	0.599	0.646
Total meat (g)	76.7 (59.0-114.3)	83.2 (53.1-115.3)	138.2 (75.0-159.2)	98.3 (74.2-135.8)	0.062	0.850	**0.031**	0.149	**0.024**	0.160	0.248
Total fish (g)	10.4 (7.0-23.8)	16.6 (9.1-20.4)	16.0 (0.0-23.8)	16.6 (12.0-27.5)	0.703	0.498	0.973	0.275	0.622	0.763	0.457
Total rest group (g)	95.3 (50.4-217.4)	197.8 (54.7-355.7)	264.7 (77.4-671.8)	209.5 (65.4-304.4)	0.199	0.551	0.057	0.190	0.150	0.672	0.201
Daily protein intake (g)	53.0 (40.1-69.3)	50.3 (42.5-60.3)	64.5 (51.1-72.4)	56.5 (47.5-67.3)	0.252	0.957	0.143	0.394	0.077	0.204	0.357
Daily fat intake (g)	38.7 (31.5-48.5)	41.7 (33.8-56.4)	53.4 (40.6-73.1)	44.7 (37.0-54.7)	0.095	0.432	**0.038**	0.114	0.077	0.448	0.157
Daily carbohydrates intake (g)	141.5 (123.9-169.8)	146.1 (120.5-180.0)	155.6 (127.8-217.2)	142.2 (120.4-178.2)	0.721	0.516	0.337	0.779	0.478	0.911	0.357
Daily fiber intake (g)	16.0 (12.9-20.1)	16.1 (13.6-18.9)	12.7 (11.4-21.1)	15.8 (12.7-18.0)	0.941	0.935	0.801	0.779	0.623	0.577	0.794
IPAQ/METs category at time of OGTT					0.981	0.736	1.000	0.861	1.000	1.000	1.000
% Low	0.00 (0)	0.00 (0)	0.00 (0)	3.85 (1)							
% Moderate	36.84 (7)	47.06 (8)	40.00 (4)	42.31 (11)							
% High	63.16 (12)	52.94 (9)	60.00 (6)	53.85 (14)							
% Clinical depression (≥16 on CES-D questionnaire)	23.81 (5)	42.11 (8)	42.86 (6)	22.22 (6)	0.322	0.314	0.283	1.000	1.000	0.199	0.278
STAI-6	12.1 ± 3.62	12.8 ± 3.75	13.4 ± 4.20	11.9 ± 3.56	0.543	0.615	0.436	0.714	0.660	0.284	0.187
% Recent changes to reduce risk	57.14 (12)	36.84 (7)	71.43 (10)	40.74 (11)	0.168	0.225	0.488	0.383	0.080	1.000	0.100
% Planning changes to reduce risk	80.95 (17)	84.21 (16)	71.43 (10)	85.19 (23)	0.740	1.000	0.658	0.715	0.422	1.000	0.411

[a]GDM, gestational diabetes mellitus; PCOS, polycystic ovary syndrome; BMI, body mass index; T2DM, type 2 diabetes mellitus.

[b]FPG, fasting plasma glucose; OGTT, oral glucose tolerance test; PPWR, postpartum weight retention; LDL-cholesterol, low density lipoprotein cholesterol; FFQ, Food Frequency Questionnaire; IPAQ, International Physical Activity Questionnaire; METs, metabolic equivalent of task [MET] minutes/week; CES-D, Center for Epidemiologic Studies–Depression; STAI-6, Spielberger State-Trait Anxiety Inventory.

[c]IFG, impaired fasting glucose; IGT, impaired glucose tolerance.

[d]The first column presents data on individuals who underestimated their risk at baseline (6-16 weeks postpartum) and continued to do so one year after randomization.

[e]The second column contains data on individuals who underestimated their risk at baseline but adequately estimated their risk one year after randomization.

[f]The third column shows data for individuals who adequately estimated their risk at baseline but underestimated their risk one year after randomization.

[g]The fourth column includes data on individuals who adequately estimated their risk at baseline and continued to do so one year after randomization.

[h,i]Categorical variables are presented as frequencies %(n); continuous variables are presented as mean ± SD if normally distributed and as median ± IQR if not normally distributed.

[j] U, underestimated the risk to develop T2DM (underestimator group: almost no chance and slight chance).

[k] A, adequately estimated the risk to develop T2DM (moderate chance and high chance).

[l]Differences are considered significant at p-value<0.05. Bold means a statistical significant value of p<0.05.

Supplementary Table S6. Control group: comparison of groups according to switch (from underestimating to adequately estimating their diabetes risk and vice versa) between baseline and one year post-randomization

	U/U (N=7, 8.5%)[d, h, I, j]	U/A (N=20, 24.4%)[e, h, I, j, k]	A/U (N=26, 31.7%)[f, h, I, j, k]	A/A (N=29, 35.4%)[g, h, I, k]	Pairwise comparison						
					**p-value** [l]	**1 vs 2**[l]	**1 vs 3**[l]	**1 vs 4**[l]	**2 vs 3**[l]	**2 vs 4**[l]	**3 vs 4**[l]
**General characteristics**[a]											
Age (years)	33.9 ± 5.46	31.5 ± 4.37	33.6 ± 4.18	32.3 ± 3.08	0.297	0.390	0.842	0.410	0.109	0.506	0.150
% Caucasian	71.43 (5)	75.00 (15)	80.77 (21)	86.21 (25)	0.677	1.000	0.623	0.573	0.726	0.456	0.721
% Non-Caucasian	28.57 (2)	25.00 (5)	19.23 (5)	13.79 (4)							
Highest education					0.733	0.738	0.376	0.227	0.727	0.595	0.920
%None/primary school	0.00 (0)	0.00 (0)	0.00 (0)	0.00 (0)							
%Until age of 15 years	0.00 (0)	5.00 (1)	3.85 (1)	10.34 (3)							
%High school	0.00 (0)	10.00 (2)	23.08 (6)	17.24 (5)							
%Higher education (bachelor/master)	100.00 (7)	85.00 (17)	73.08 (19)	72.41 (21)							
% Paid professional activity	85.71 (6)	90.00 (18)	73.08 (19)	89.66 (26)	0.342	1.000	0.652	1.000	0.262	1.000	0.164
Monthly net income family					0.504	0.756	0.190	0.244	0.433	0.570	1.000
%Low income <1500	14.29 (1)	5.00 (1)	3.85 (1)	3.45 (1)							
%€1500-5000	71.43 (5)	80.00 (16)	92.31 (24)	89.66 (26)							
% >€5000	14.29 (1)	15.00 (3)	3.85 (1)	6.90 (2)							
% Living without partner	14.29 (1)	15.00 (3)	19.23 (5)	20.69 (6)	0.973	1.000	1.000	1.000	1.000	0.720	1.000
% Currently smoking	0.00 (0)	0.00 (0)	15.38 (4)	6.90 (2)	0.292	0.137	0.066	1.000	0.758	**0.007**	**0.003**
% Multiparity	85.71 (6)	55.00 (11)	38.46 (10)	62.07 (18)	0.106	0.148	**0.026**	0.234	0.264	0.621	0.080
% History of GDM in previous pregnancy	33.33 (2)	21.43 (3)	20.00 (3)	18.18 (4)	0.881	0.573	0.517	0.423	0.924	0.810	0.890
% History of PCOS	0.00 (0)	5.56 (1)	4.17 (1)	0.00 (0)	0.617	0.524	0.583	-	0.834	0.207	0.275
% History of miscarriage	57.14 (4)	45.00 (9)	38.46 (10)	37.93 (11)	0.787	0.580	0.375	0.355	0.655	0.621	0.968
Pre-pregnancy BMI (kg/m²)	26.9 ± 5.43	25.2 ± 5.25	26.6 ± 5.96	29.7 ± 4.88	**0.026**	0.543	0.947	0.280	0.471	**0.005**	**0.023**
% First degree family history of T2DM	16.67 (1)	16.67 (3)	42.31 (11)	39.29 (11)	0.222	1.000	0.242	0.293	0.073	0.104	0.821
% Second degree family history of T2DM	33.33 (2)	52.94 (9)	65.22 (15)	81.82 (18)	0.092	0.408	0.158	**0.020**	0.433	0.053	0.208
**Baseline (6-16 weeks postpartum)**[b]											
FPG (mmol/L)	5.25 ± 0.54	5.14 ± 0.55	5.17 ± 0.60	5.48 ± 0.55	0.177	0.657	0.877	0.401	0.947	0.053	0.070
Glycemia 60 min (mmol/L)	8.67 ± 0.71	9.62 ± 2.07	10.05 ± 2.08	9.03 ± 1.66	0.062	0.135	**0.029**	0.379	0.210	0.445	**0.037**
Glycemia 120 min (mmol/L)	7.94 ± 1.06	8.07 ± 1.58	8.66 ± 0.95	7.75 ± 1.34	0.068	0.803	0.074	0.952	0.169	0.483	**0.011**
Timing OGTT (weeks)	11.6 ± 3.25	13.1 ± 2.88	12.2 ± 2.24	13.0 ± 2.32	0.356	0.332	0.567	0.298	0.223	0.968	0.145

BMI (kg/ m²)	27.6 ± 5.39	25.7 ± 5.80	26.9 ± 5.84	29.8 ± 4.85	**0.045**	0.455	0.775	0.448	0.419	**0.005**	0.060
Mean systolic blood pressure (mmHg)	118.1 ± 12.71	119.6 ± 15.02	119.6 ± 11.99	118.2 ± 11.11	0.959	0.825	0.724	0.936	0.723	0.976	0.642
Mean diastolic blood pressure (mmHg)	71.0 ± 14.76	75.6 ± 12.50	74.0 ± 9.65	76.5 ± 8.53	0.560	0.376	0.427	0.237	0.765	0.575	0.340
% Hypertension	14.29 (1)	15.00 (3)	11.54 (3)	6.90 (2)	0.822	0.963	0.843	0.526	0.730	0.357	0.550
Waist circumference (cm)	93.4 ± 7.26	88.8 ± 14.49	90.4 ± 12.93	99.2 ± 12.48	**0.019**	0.286	0.469	0.168	0.649	**0.010**	**0.011**
% Waist circumference >80 cm	100.00 (6)	70.00 (14)	76.92 (20)	96.30 (26)	**0.049**	0.126	0.192	0.632	0.596	**0.012**	**0.037**
PPWR (kg)	1.9 ± 6.11	1.2 ±4.22	1.1 ± 4.83	0.5 ± 5.64	0.988	0.825	0.775	0.810	0.816	0.903	1.000
PPWR > 0 kg	57.14 (4)	50.00 (10)	57.69 (15)	55.17 (16)	0.962	0.745	0.979	0.925	0.604	0.721	0.851
PPWR > 5kg	14.29 (1)	15.00 (3)	15.38 (4)	13.79 (4)	0.999	0.963	0.943	0.973	0.971	0.906	0.867
HbA1c (%)	5.3 ± 0.38	5.4 ± 0.36	5.4 ± 0.41	5.4 ± 0.41	0.915	0.978	0.707	0.984	0.867	0.653	0.499
HbA1c (mmol/mol)	34.9 ± 4.13	35.5 ± 3.94	35.9 ± 4.50	35.2 ± 4.45	0.915	0.978	0.707	0.984	0.867	0.653	0.499
Fasting LDL-cholesterol (mmol/L)	3.10 ± 0.99	2.73 ± 0.68	3.11 ± 0.85	3.02 ± 0.92	0.623	0.524	0.809	0.967	0.183	0.385	0.716
FFQ:											
Total water (g)	2199.1 (1800.7-2655.3)	2189.6 (1434.0-2427.7)	1999.5 (1723.3-2364.0)	2284.1 (1945.1-2481.6)	0.633	0.489	0.441	0.842	0.973	0.399	0.270
Total fruit (g)	32.2 (17.2 – 120.0)	120.0 (41.8-225.0)	120.0 (17.2-225.0)	96.5 (51.5-225.0)	0.683	0.404	0.393	0.149	0.946	0.820	0.931
Total vegetables (g)	240.0 (94.3-240.0)	240.0 (120.0-240.0)	214.3 (103.0-240.0)	188.6 (120.0-240.0)	0.936	0.836	1.000	0.636	0.720	0.966	0.574
Total meat (g)	135.3 (53.8-202.4)	87.1 (62.8-138.6)	94.3 (68.2-136.7)	117.2 (91.1-141.6)	0.227	0.580	0.708	0.984	0.833	0.070	0.067
Total fish (g)	29.7 (16.6-47.6)	17.7 (10.1-30.4)	16.2 (8.6-24.7)	12.9 (9.6-22.2)	0.193	0.268	0.078	**0.045**	0.492	0.263	0.673
Total rest group (g)	434.7 (20.3-479.7)	219.0 (93.1-387.4)	134.3 (86.0-409.3)	348.9 (148.7-451.5)	0.644	0.890	0.676	0.936	0.542	0.376	0.235
Daily protein intake (g)	61.1 (57.4-103.1)	61.1 (57.4-103.1)	58.9 (49.9-112.3)	65.7 (53.1-74.9)	0.642	0.281	0.271	0.472	0.938	0.576	0.505
Daily fat intake (g)	51.8 (50.8-70.8)	54.2 (43.2-68.2)	47.5 (37.7-59.9)	57.7 (48.2-67.1)	0.501	0.761	0.194	0.795	0.346	0.753	0.222
Daily carbohydrates intake (g)	213.9 (184.1-235.7)	206.9 (157.9-235.4)	174.7 (146.9-199.5)	180.6 (156.6-214.2)	0.318	0.525	0.075	0.129	0.303	0.535	0.631
Daily fiber intake (g)	20.2 (17.1-21.6)	17.6 (15.2-20.9)	17.7 (11.2-19.4)	18.2 (13.6-22.6)	0.510	0.234	0.118	0.436	0.650	0.879	0.453
IPAQ/METs category at time of OGTT					0.952	1.000	0.838	1.000	0.908	0.850	0.696
% Low	16.67 (1)	10.53 (2)	12.50 (3)	17.86 (5)							
% Moderate	33.33 (2)	42.11 (8)	50.00 (12)	35.71 (10)							
% High	50.00 (3)	47.37 (9)	37.50 (9)	46.43(13)							
STAI-6	10.4 ± 2.57	11.3 ± 3.37	13.6 ± 3.36	11.2 ± 2.54	**0.013**	0.801	**0.031**	0.628	**0.014**	0.821	0.005
**One year after randomization[c]**											
% Breastfeeding	71.43 (5)	95.00 (19)	76.92 (20)	75.86 (22)	0.227	0.156	1.000	1.000	0.119	0.119	1.000
FPG (mmol/L)	5.49 ± 0.61	5.38 ± 0.61	5.34 ± 1.00	5.44 ± 0.58	0.650	0.868	0.427	0.826	0.438	0.862	0.234
Glycemia 60 min (mmol/L)	7.76 ± 1.86	8.67 ± 2.21	9.20 ± 2.72	8.59 ± 2.22	0.494	0.346	0.094	0.337	0.557	0.943	0.516
Glycemia 120 min (mmol/L)	6.64 ± 1.27	7.09 ± 1.50	7.69 ± 3.01	7.49 ± 1.55	0.494	0.472	0.481	0.215	0.748	0.234	0.458
Timing OGTT (weeks)	66.7 ± 5.74	65.0 ± 3.39	65.9 ± 3.30	65.7 ± 3.51	0.812	0.802	1.000	0.872	0.337	0.467	0.722
% T2DM	0.00 (0)	5.00 (1)	11.54 (3)	0.00 (0)	0.228	0.547	0.346	-	0.435	0.224	0.060
% IFG	60.00 (3)	40.00 (4)	9.09 (1)	25.00 (5)	0.353	0.526	0.053	0.217	0.206	0.674	0.495

% IGT	40.00 (2)	40.00 (4)	45.45 (5)	45.00 (9)	0.353	0.526	0.053	0.217	0.206	0.674	0.495
% IFG + IGT	0.00 (0)	20.00 (2)	45.45 (5)	30.00 (6)	0.353	0.526	0.053	0.217	0.206	0.674	0.495
BMI (kg/ m²)	26.4 ± 4.10	25.9 ± 6.70	26.0 ± 6.52	29.6 ± 4.87	**0.034**	0.698	0.947	0.631	0.506	0.148	0.710
Mean systolic blood pressure (mmHg)	118.1 ± 9.26	116.8 ± 9.63	118.1 ± 16.65	120.9 ± 11.75	0.622	0.360	0.233	0.173	0.641	0.632	1.000
Mean diastolic blood pressure (mmHg)	72.0 ± 7.57	76.0 ± 10.29	77.3 ± 9.67	77.3 ± 8.83	0.569	0.903	0.281	0.072	0.126	**0.025**	0.772
% Hypertension	0.00 (0)	20.00 (4)	19.23 (5)	6.90 (2)	0.302	0.200	0.208	0.475	0.948	0.169	0.171
Waist circumference (cm)	89.3 ± 10.60	85.1 ± 14.75	86.1 ± 18.91	95.3 ± 11.40	**0.014**	0.394	0.346	0.293	1.000	**0.010**	**0.005**
% Waist circumference >80 cm	66.67 (4)	30.00 (6)	38.46 (10)	72.41 (21)	0.054	0.940	0.687	0.143	0.615	**0.035**	**0.007**
PPWR (kg)	-1.4 ± 10.41	1.8 ± 6.02	-1.4 ± 5.43	-0.2 ± 4.66	0.515	0.618	0.792	0.889	0.138	0.404	0.414
PPWR > 0 kg	42.88 (3)	55.00 (11)	42.31 (11)	48.28 (14)	0.850	0.580	0.979	0.797	0.393	0.644	0.657
PPWR > 5kg	14.29 (1)	25.00 (5)	7.69 (2)	13.79 (4)	0.433	0.557	0.590	0.973	0.105	0.319	0.469
HbA1c (%)	5.3 ± 0.47	5.3 ± 0.34	5.4 ± 0.57	5.4 ± 0.35	0.733	0.845	0.863	0.763	0.834	0.225	0.499
HbA1c (mmol/mol)	34.7 ± 5.15	34.2 ± 3.69	35.7 ± 6.26	35.0 ± 3.86	0.733	0.845	0.863	0.763	0.834	0.225	0.499
Fasting LDL-cholesterol (mmol/L)	3.24 ± 0.88	2.84 ± 0.85	2.84 ± 0.87	2.93 ± 0.79	0.641	0.333	0.322	0.353	0.825	0.451	0.615
FFQ:											
Total water (g)	1903.4 (1452.9-2156.7)	2157 (1663.6-2452.0)	1724.9 (1418.9-2281.2)	2323.2 (1691.3-2593.6)	0.081	0.525	0.467	0.230	0.195	0.250	**0.016**
Total fruit (g)	96.5 (32.2-176.9)	95.4 (24.7-225.0)	108.3 (51.5-225.0)	96.5 (96.5-176.9)	0.803	0.933	0.655	0.417	0.812	0.451	0.578
Total vegetables (g)	240.0 (188.6-240.0)	240.0 (120.0-240.0)	240.0 (120.0-240.0)	188.6 (120.0-240.0)	0.657	0.658	0.400	0.164	0.713	0.490	0.877
Total meat (g)	106.1 (75.6-139.5)	82.0 (59.9-137.6)	75.3 (62.9-131.9)	100.1 (71.4-149.0)	0.549	0.376	0.344	0.857	0.947	0.276	0.312
Total fish (g)	18.5 (16.6-24.7)	15.0 (11.4-24.2)	14.8 (9.6-20.4)	10.4 (8.6-18.5)	0.203	0.421	0.226	0.114	0.542	0.108	0.265
Total rest group (g)	329.9 (33.1-500.4)	158.1 (47.9-468.3)	118.4 (50.4-524.4)	345.3 (167.8-447.9)	0.625	0.978	0.912	0.576	0.973	0.286	0.248
Daily protein intake (g)	62.5 (44.6-74.2)	53.6 (41.7-65.8)	54.7 (40.2-63.0)	55.1 (50.4-66.8)	0.635	0.422	0.243	0.719	0.698	0.522	0.390
Daily fat intake (g)	44.3 (29.0-65.0)	45.1 (38.7-57.5)	51.3 (34.4-60.5)	50.7 (39.2-57.2)	0.851	0.761	0.467	0.472	0.587	0.722	0.833
Daily carbohydrates intake (g)	188.8 (158.6-220.9)	162.9 (134.8-187.8)	142.8 (117.4-189.5)	183.1 (130.0-209.9)	0.194	0.143	**0.036**	0.472	0.587	0.344	0.180
Daily fiber intake (g)	18.8 (16.2-21.8)	16.0 (11.8-20.7)	14.7 (12.6-18.3)	17.3 (12.7-21.2)	0.223	0.158	**0.026**	0.337	0.634	0.590	0.241
IPAQ/METs category at time of OGTT					0.502	0.807	0.613	0.522	0.164	0.597	0.539
% Low	20.00 (1)	22.22 (4)	8.70 (2)	12.00 (3)							
% Moderate	20.00(1)	44.44 (8)	30.43 (7)	44.00 (11)							
% High	60.00(3)	33.33(6)	60.87 (14)	44.00 (11)							
% Clinical depression (≥16 on CES-D questionnaire)	0.00 (0)	15.00 (3)	38.46 (10)	13.79 (4)	0.061	0.545	0.073	0.566	0.106	1.000	0.061
STAI-6	11.4 ± 3.51	12.3 ± 3.40	13.3 ± 3.77	12.6 ± 3.49	0.697	0.675	0.257	0.560	0.426	0.886	0.519
% Recent changes to reduce risk	28.57 (2)	60.00 (12)	50.00 (13)	38.62 (17)	0.512	0.209	0.413	0.219	0.561	1.000	0.593
% Planning changes to reduce risk	42.86 (3)	95.00 (19)	61.54 (16)	79.31 (23)	**0.010**	**0.009**	0.422	0.076	**0.013**	0.216	0.234

[a]GDM, gestational diabetes mellitus; PCOS, polycystic ovary syndrome; BMI, body mass index; T2DM, type 2 diabetes mellitus.

[b]FPG, fasting plasma glucose; OGTT, oral glucose tolerance test; PPWR, postpartum weight retention; LDL-cholesterol, low density lipoprotein cholesterol; FFQ, Food Frequency Questionnaire; IPAQ, International Physical Activity Questionnaire; METs, metabolic equivalent of task [MET] minutes/week; CES-D, Center for Epidemiologic Studies–Depression; STAI-6, Spielberger State-Trait Anxiety Inventory.

[c]IFG, impaired fasting glucose; IGT, impaired glucose tolerance.

[d]The first column presents data on individuals who underestimated their risk at baseline (6-16 weeks postpartum) and continued to do so one year after randomization.

[e]The second column contains data on individuals who underestimated their risk at baseline but adequately estimated their risk one year after randomization.

[f]The third column shows data for individuals who adequately estimated their risk at baseline but underestimated their risk one year after randomization.

[g]The fourth column includes data on individuals who adequately estimated their risk at baseline and continued to do so one year after randomization.

[h,i]Categorical variables are presented as frequencies %(n); continuous variables are presented as mean ± SD if normally distributed and as median ± IQR if not normally distributed.

[j] U, underestimated the risk to develop T2DM (underestimator group: almost no chance and slight chance).

[k] A, adequately estimated the risk to develop T2DM (moderate chance and high chance).

[l]Differences are considered significant at p-value<0.05. Bold means a statistical significant value of p<0.05.
